# Driving forces shaping the microbial ecology in meat packing plants

**DOI:** 10.3389/fmicb.2023.1333696

**Published:** 2024-01-23

**Authors:** Xianqin Yang, Claudia Narvaez-Bravo, Peipei Zhang

**Affiliations:** ^1^Lacombe Research and Development Centre, Agriculture and Agri-Food Canada, Lacombe, AB, Canada; ^2^Food and Human Nutritional Sciences, University of Manitoba, Winnipeg, MB, Canada; ^3^Department of Animal Sciences, Center for Meat Safety and Quality, Colorado State University, Fort Collins, CO, United States

**Keywords:** microbiota, meat processing facilities, beef, pork, persistent bacteria, sequencing, Shiga toxin-producing *Escherichia coli*, biofilm

## Abstract

Meat production is a complex system, continually receiving animals, water, air, and workers, all of which serve as carriers of bacteria. Selective pressures involved in different meat processing stages such as antimicrobial interventions and low temperatures, may promote the accumulation of certain residential microbiota in meat cutting facilities. Bacteria including human pathogens from all these sources can contaminate meat surfaces. While significant advancements have been made in enhancing hygienic standards and pathogen control measures in meat plants, resulting in a notable reduction in STEC recalls and clinical cases, STEC still stands as a predominant contributor to foodborne illnesses associated with beef and occasionally with pork. The second-and third-generation sequencing technology has become popular in microbiota related studies and provided a better image of the microbial community in the meat processing environments. In this article, we reviewed the potential factors influencing the microbial ecology in commercial meat processing facilities and conducted a meta-analysis on the microbiota data published in the last 10 years. In addition, the mechanisms by which bacteria persist in meat production environments have been discussed with a focus on the significant human pathogen *E. coli* O157:H7 and generic *E. coli*, an indicator often used for the hygienic condition in food production.

## 1 Introduction

The process of converting livestock to wholesale/retail-ready packages of meat products in commercial practice is complex and systematic. Meat processing facilities are open systems in that there is a constant intake of animals, water, air, and workers, all of which serve as carriers of bacteria (Cobo-Díaz et al., 2021). On the other hand, certain locations in the facilities may be conducive to the accumulation of residential microbiota, due to the selective pressures associated with the microenvironment that favors subgroups of the incoming microbiota. During the meat production process, bacteria carried by animals at slaughter and from the processing facility environment can be deposited on meat surfaces, a fraction of which may be pathogenic. For example, STEC does not cause overt disease in cattle but is a significant human pathogen, with some strains having an infectious dose as low as a few cells (Thorpe, 2004). STEC infections can lead to symptoms ranging from mild gastrointestinal discomfort to more serious conditions such as bloody diarrhea and, in some cases, life-threatening complications like hemolytic uremic syndrome (HUS), particularly for vulnerable populations such as young children, the elderly, and individuals with compromised immune systems (Tarr et al., 2005; CDC, 2014). In North America, 7 serogroups of *E. coli*, namely O157, O103, O26, O111, O121, O45, and O145, are more frequently associated with severe clinical outcomes than other serogroups and are thus referred to as Top 7 *E. coli* (Heiman et al., 2015). Beef is the most common vehicle in STEC transmission in North America (WHO, 2018). In recent years, there have also been reports on STEC in pork processing plants in North America (Essendoubi et al., 2020; Haque et al., 2022). To reduce microbial contamination and ensure the safety of meat, antimicrobial interventions are routinely implemented in meat processing facilities. Even though the measures are intended to target pathogenic bacteria, they inevitably have consequences on commensal microorganisms, due to the broad spectrum of their activities (Youssef et al., 2014). In addition to biocides, common operational conditions such as low temperatures and desiccation from drying equipment or carcass surfaces in meat processing facilities also play a role in shaping the microbial ecology in such environments. The wide adoption of various sequencing tools has resulted in a much higher resolution of the microbial population structure. The present work aimed to review factors shaping the microbial ecology in commercial meat processing facilities, the bacteria persisting in meat production environment with a focus on microbiome data published in the last 10 years (2013–2023), and the mechanisms applied by bacteria to persist.

## 2 Carriage of bacteria by animals

The gastrointestinal (GI) tracts of mammalians harbor trillions of microorganisms. It is estimated that the bacterial populations of cattle and pigs’ intestines often exceed 10^11^ CFU/gram feces (Gaskins et al., 2002; Dowd et al., 2008). Not only are they high in number, but also these bacterial communities are very diverse. In a study of Mao et al. (2015) more than 542 genera belonging to 23 phyla were found in the GI tracts of cattle, with *Prevotella*, *Treponema*, *Succiniclasticum, Ruminococcus*, *Acetitomaculum*, *Mogibacterium*, *Butyrivibrio* and *Acinetobacter*, as well as those unclassified on genus level but derived from higher taxonomic ranks *Peptostreptococcaceae*, *Ruminococcaceae*, *Enterobacteriaceae*, *Prevotellaceae*, *Clostridiales*, *Rikenellaceae* and *Bacteroidales* being most abundant. Similarly, a shotgun metagenomic analysis of pig feces revealed 2,797 bacterial genera, with the most abundant ones being *Clostridium*, *Clostridioides*, *Escherichia*, *Prevotella*, *Bacteroides*, and *Treponema* (Quan et al., 2020).

Both husbandry practices and the inherent host factors of animals affect the gut microbiome (Bessegatto et al., 2017; Zhang et al., 2021; Lin et al., 2022). A change in environment especially those eliciting stressors also affects the gut microbiome. Fecal shedding of *E. coli* O157 often increases in summer seasons as reviewed by Kempf et al. (2022). Placing animals in a feedlot decreased the diversity in individual animals and the similarity between animals after 2 days, but these changes diminished at 7 and 14 days after the placement, suggesting the native microbiome in a cattle tends to be robust (Maslen et al., 2022). However, changes in minor or rare taxa may not always be sufficiently captured by metagenomic based studies. For example, when considering cattle exhibiting a fecal carriage of *E. coli* O157 at a concentration of 10^4^ CFU/g, they are classified as “super shedders” (Stephens et al., 2009). However, even at this high shedding level, *E. coli* O157 remains a minute fraction within the vast landscape of the total fecal bacterial load, which typically reaches 10^11^ CFU/g. In addition, it is also challenging to assemble metagenomes of different strains of the same species in a diverse microbial population (Meziti et al., 2021). Khaitsa and co-workers examined the fecal shedding of naturally occurring *E. coli* O157:H7 in steers from the same calf cohort and fed the same diet, by culture-dependent methods (Khaitsa et al., 2003). They found the prevalence of *E. coli* O157:H7 varied with sampling time for animals in the same pen and varied among pens at the same sampling time, both from 0% to 100%. To capture these changes and have a holistic picture of the microbiome, some form of enrichment would be necessary.

The bacteria on a particular animal hide could originate from feces of animals sharing the same space, through cross-contamination from fecal origin but are better equipped to survive in secondary environments other than the host, and from the rearing environments such as soil, bedding, water etc (Narvaez-Bravo et al., 2013). The fecal microbiota are primarily mesophilic microorganisms, and can be very different from those that inhabits the animal rearing environments (Zaheer et al., 2019). In contrast with the commonly found 10^11^ CFU/gram feces bacterial load in cattle intestines, the number of bacteria on hides varies largely, from 10^4^ to 10^10^ CFU/cm^2^ in various studies from several countries and with different ratios of *Enterobacteriaceae* and coliforms to the total bacterial population, likely reflecting the differences in climate and animal husbandry practices (Yang, 2017). A systematic review and meta-analysis of the prevalence and concentration of *E. coli* O157 in cattle in North America showed an overall fecal prevalence of 10.7% and hide prevalence at processing plants of 56.4% (Ekong et al., 2015). The percentage of cattle carrying high concentration of *E. coli* O157:H7 on hides was much higher at processing plants (23.81%) than at feedlots (1.74%) for fed beef, likely resulting from much closer contact between animals and stress during transportation. Even so, different and diverse genotypes as determined by pulse field gel electrophoresis were observed for *E. coli* O157 on hides of cattle processed on the same day and on different days at the same processing facility (Arthur et al., 2014), suggesting the microbial diversity on hide of incoming animals is also reflected by within species diversity.

## 3 Meat packing process

### 3.1 Process

The process of converting cattle to meat products in commercial slaughter plants can involve more than 50 different operations (Supplementary Table S1), which can be divided into four major stages: slaughtering of animals, carcass dressing, carcass chilling and carcass breaking (Yang, 2017). Most packing plants perform the operations in that order, however, carcass breaking can also happen before carcass is completely chilled, through a hot boning process (Keenan et al., 2016). Food safety programs such as standard sanitation procedures, good manufacturing practices and hazard analysis critical control point (HACCP) systems are implemented especially in state-inspected processing facilities by which microbiological effects of operations are analyzed and control measures implemented to minimize such effects (Gill, 2005). The microbiological impact of slaughtering (stunning and bleeding) on meat can be negligible as contamination is localized around the wound. Muscle tissues of healthy animals are literally free of bacteria; however, microorganisms will be introduced to the previously sterile meat surface upon exposure. For instance, the initial cuts of the skinning process where knives work from outside to inside of the skin and bioaerosols generated by flapping of the hide during the dehiding operations inevitably deposit bacteria on carcass surfaces. Leakage of gut contents could also serve as sources of contamination. However, with improvement in dressing operations in modern meat processing facilities, gut leakage has become a rare event during the removal of viscera (Blagojevic et al., 2012). Most bacteria contaminating skinned carcass surfaces came from the hide of animals (Arthur et al., 2004). Pork dressing can differ from that for cattle in that it includes scalding, dehairing, singeing and polishing (black scraping) of carcasses before evisceration and the dressing process does not always involve hide/skin removal (Nastasijevic et al., 2020).

Temperature plays a crucial role in microbial growth and can significantly impact the rate at which microorganisms proliferate. Temperature can affect enzymatic activity and membrane fluidity leading to reduced cellular activity. The minimum growth temperature for *E. coli* and associated mesophilic organisms is around 8°C (Shaw et al., 1971; Smith, 1985). Regulatory agencies in some countries require the surface temperature of carcasses (beef and pork) be reduced to 7°C or below within 24 h of carcass dressing, and an internal temperature (the warmest part) of 7°C be reached before further processing (CFIA, 2010). In commercial practice, carcasses are chilled either by spray chilling where carcasses are intermittently sprayed with water, or by dry chilling where they are exposed to a flow of refrigerated air (Savell et al., 2005). Air chilling with appropriate parameters can be an effective antimicrobial intervention step, resulting in >2 log reductions of the carcass bacterial population, likely by the desiccating effect from the rapid evaporation of water from warm carcass surfaces in a refrigerated environment (Greig et al., 2012; Liu et al., 2016; Zdolec et al., 2022). In contrast, no consistent antimicrobial effects have been reported for spray chilling, with increase, decrease and no change in bacterial numbers on carcass surfaces have been reported (Yang, 2017). *Psychrobacter* and *Pseudomonas*, respectively, were the dominant taxa among the bacterial population on spray chilled and dry chilled carcasses, likely resulting from differences in carcass surface water activity by the two chilling methods (Yang et al., 2017b).

Carcass breaking is the process by which carcass sides are made into cuts with predetermined specifications, and trimmed off pieces (trimmings) are collected into large containers. At large facilities, the process can be very complex, involving the progressive removal of portions from the hanging side and each portion being passed to a separate collection or a processing line. In this process, multiple tools such as saws, knives, cutting boards and conveyor belts are used and workers often wear gloves (cotton, mesh and or plastic), aprons and other protective gears. The air temperature at fabrication facility is maintained at below 10°C to minimize growth of mesophilic bacteria (CFIA, 2010).

### 3.2 Antimicrobial interventions

In spite of the potential sources of bacterial contamination described above, antimicrobial interventions implemented in commercial plants can result in significant reductions of the bacterial populations and may also affect the composition of the microbial populations. The commonly used antimicrobial interventions in beef processing facilities during dressing process include hide-on carcass wash with NaOH (Yang et al., 2015b), spraying carcasses (pre-evisceration) and carcass sides (post-evisceration) with solutions of lactic acid, acetic acid, peroxyacetic acid or organic acid mixtures and pasteurization of carcass sides with hot water or steam (Algino et al., 2007; Gill, 2009; Yang et al., 2012; Scott et al., 2015). Spraying carcasses with ozone has also been reported (Casas et al., 2021). The application of spray/wash is carried out either in large spray cabinets through which carcass sides sequentially pass or using small handheld sprayers, depending on the line speed, space availability and cost. Even for the same antimicrobial intervention type, different application parameters such as concentrations of chemicals, the way the spray is applied, water temperature as well the topographical/physiochemical properties of the treatment surface may vary from study to study and consequently their antimicrobial efficacies. A meta-analysis of popular interventions used in cattle processing plants to reduce *E. coli* contamination revealed that the initial microbial concentrations and timing of extra water washes were the most important predictors of intervention effectiveness (Zhilyaev et al., 2017). The mean reduction of *E. coli* on hides by water, acetic acid, lactic acid, and sodium hydroxide wash were 0.08, 2.21, 3.02, 3.66 log units, while for carcasses, the mean reductions by water, acetic acid and lactic acid were 1.90, 1.44, and 2.07 log units. In addition, applications toward targeted areas such as trimming, vacuum cleaning with or without spraying with hot water, spraying the initial opening cut lines with lactic acid are also in use. Their effects on the microbiological conditions of carcasses would be localized (Gill, 2009). Intermittent application of low concentrations of oxidizing agents such as chlorine and peroxyacetic acid during spray chilling showed up to 4-log reductions of both *Salmonella* and *E. coli* in laboratory settings (Kocharunchitt et al., 2020), likely resulting from the synergistic effect of low temperature and oxidative stress on these enteric pathogens (King et al., 2016). In some facilities, cuts and trimmings are misted with 200 ppm peroxyacetic acid over the conveyor belt through the spray cabinet (Yang et al., 2012, 2021). This treatment is ineffective for reducing the number of bacteria on meat surface but may affect the survival and growth of subpopulations during extended storage under chilled and vacuum packaged conditions. In addition to antimicrobial interventions, bacteria on pork carcasses during dressing can also be significantly reduced by scalding and singeing but not by polishing or dehairing, all of which are unique to pork carcass dressing (Zdolec et al., 2022).

### 3.3 Fabrication equipment

It is commonly believed that bacteria on meat products are primarily from chilled carcasses and contribution from other sources, if at all, would be negligible. If so, the number of bacteria on meat products would be lower than that on chilled carcass surface, with the area extension of newly cut surfaces during the fabrication process. A study of Youssef et al. shows that the numbers of aerobes and *E. coli* on cuts and trimmings are >10 times the number for chilled carcasses and the number of coliforms on products are >100 times the number for chilled carcasses (Youssef et al., 2013). Genotyping of *E. coli* on beef products, incoming chilled carcasses and surfaces of various equipment involved with fabrication has found the majority of *E. coli* on products share the same genotype with *E. coli* on equipment surfaces, rather than chilled carcasses (Yang et al., 2015a, 2017a). The same genotypes of STEC O157:H7 across “high-event periods” over time and of generic *E. coli* on beef trimmings produced on different days at the same processing facility have been reported (Arthur et al., 2014; Visvalingam et al., 2016). In addition, some *E. coli* strains persisted, evidenced by repeated recovery of the same genotype on multiple sampling times from equipment at the same facility (Yang et al., 2017c). Even though processing facilities and equipment involved with meat fabrication are cleaned and sanitized daily, the goal of sanitation is to achieve visibly clean equipment rather than sterility of equipment, and standards on maximum number of bacteria on post sanitization are often lacking.

## 4 A meta-analysis of persistent bacteria in meat processing environment

As we have discussed above, the meat processing environment, e.g., fabrication equipment surfaces, is an important source of bacteria contaminating meat. We performed a meta-analysis on the potential persistent microbiota in meat production environment. Literature search was conducted in Google Scholar and Scopus with “microbiota”/“microbiome”/“metagenomics”/“sequencing” and “meat processing/cutting plant” as keywords. The literature which was published between 2013 and 2023 and investigated the microbiota in meat production environment using high-throughput sequencing technology was used in the analysis in the present study.

A total of 22 relevant references were found (Supplementary Table S2). In these studies, metagenome DNA, 16S rRNA gene DNA or RNA using long- or short-read sequencing technology were sequenced, mainly identifying bacteria at the genus level. We were able to retrieve sequencing data from 10 of these studies. The raw sequencing reads for relevant samples were downloaded from NCBI^1^ using Faster-dump of SRA toolkit v3.0.3.^2^

### 4.1 Data processing

For the 16S rRNA gene amplicon of a hypervariable region sequenced under Illumina platform, the primers in sequencing reads were removed using Cutadapt v4.4 (Martin, 2011). For the sequencing of full-length 16S rRNA gene amplicon using PacBio platform, the primers were removed using removePrimers function of Dada2 v1.26.0 in R v4.2.3 (Callahan et al., 2016). Dada2 workflow was used to process trimmed reads. Both forward and reverse reads were, respectively, truncated to 220 and 220 bases for Illumina sequencing. The reads < 1,000 bp for PacBio sequencing were removed. Taxa of sequencing reads were assigned using SILVA 16S rRNA gene database (v138.1; Quast et al., 2013).

For metagenomics sequencing data, Fastp v0.23.2 was used to remove adapters and bases with mean quality score < 15 at the beginning and end of the reads (Chen et al., 2018). Bowtie2 v2.5.1 was used to remove Phix contamination and animal DNA contamination with Phix (accession in NCBI, NC_001422.1) and cattle (GCA_002263795.2) or pig (GCA_000003025.6) genomes as references (Langmead and Salzberg, 2012). Taxonomy assignment for sequencing reads was performed using Kraken2 v1.1.1 and improved using Bracken v2.8 (Lu et al., 2017; Wood et al., 2019). The output was converted to Biom format using Kraken-biom v1.2.0^3^ and imported into R using package Phyloseq v1.30.0 (McMurdie and Holmes, 2013).

The relative abundance of taxa in each sample was summarized and plotted using Phyloseq and ggplot2 v3.4.2 (Wickham, 2016). The alpha and beta diversity were analyzed using both Phyloseq and Vegan v2.6.4.^4^

We used the sequencing data for samples collected from various meat plant environmental surfaces after cleaning and/or sanitation to identify persistent bacteria. A total of 447 samples collected from environmental surfaces such as conveyor belt, chopping board, equipment surfaces and other surfaces at eight meat processing plants were included (Kang et al., 2019, 2020; Botta et al., 2020; Cobo-Díaz et al., 2021; Cherifi et al., 2022; Yang et al., 2023b; Table 1; Supplementary Table S3). These plants were, respectively, located in geographical regions including Canada (Cherifi et al., 2022; Yang et al., 2023b), Italy (Botta et al., 2020), Spain (Cobo-Díaz et al., 2021), and Australia (Kang et al., 2019, 2020). One functional area/room was investigated in most of these plants except for the plant in Spain (Cobo-Díaz et al., 2021), in which four rooms including pork carcass chilling room, pork cutting room, pork packing or storage room, and Trotter’s washing room were studied. For the convenience of data presentation, we assigned labels for the rooms based on their function and origin. There were 9 rooms in total (RA01-RA09; Table 1).

**Table 1:** The functional meat processing area/rooms included in the analysis.

Room ID	Sequencing target	Room function	Reference	Country	Number of samples	Plant number
RA01	Metagenome	Pork carcass chilling	Cobo-Díaz et al. (2021)	Spain	4	PA1
RA02	Metagenome	Pork cutting	Cobo-Díaz et al. (2021)	Spain	14	PA1
RA03	Metagenome	Pork packing or storage	Cobo-Díaz et al. (2021)	Spain	15	PA1
RA04	Metagenome	Trotter’s washing room	Cobo-Díaz et al. (2021)	Spain	6	PA1
RA05	V3-V4 region of 16S rRNA gene	Beef cutting	Botta et al. (2020)	Italy	194	PA2
RA06	V4 region of 16S rRNA gene	Beef cutting	Kang et al. (2020)	Australia	80	PA3
RA07	V4 region of 16S rRNA gene	Beef cutting	Yang et al. (2023a,b)	Canada	9	PA4
RA08	V4 region of 16S rRNA gene	Beef slaughter	Kang et al. (2019)	Australia	80	PA5
RA09	V4 region of 16S rRNA gene	Pork cutting	Cherifi et al. (2022)	Canada	45	PA6
RB01	Full-length 16S rRNA gene	Pig slaughter	Zwirzitz et al. (2020)	Austria	21	PB1
RB02	Full-length 16S rRNA gene	Pork cutting	Zwirzitz et al. (2021)	Austria	27	PB1
RB03	Metagenome	Beef cutting	Yang et al. (2016)	USA	8	PB2
RB04	V3-V4 region of 16S rRNA gene	Beef cutting	Botta et al. (2020)	Italy	91	PB3
RB05	V4 region of 16S rRNA gene	Pig slaughter	Shedleur-Bourguignon et al. (2023)	Canada	292	PB4

“RA**” or “PA*” represent the functional rooms or plants for samples collected after cleaning/sanitation, while “RB**” or “PB*” represent those for samples collected before cleaning/sanitation.

### 4.2 Predominant persistent bacteria in meat processing plant

**Figure 1:**
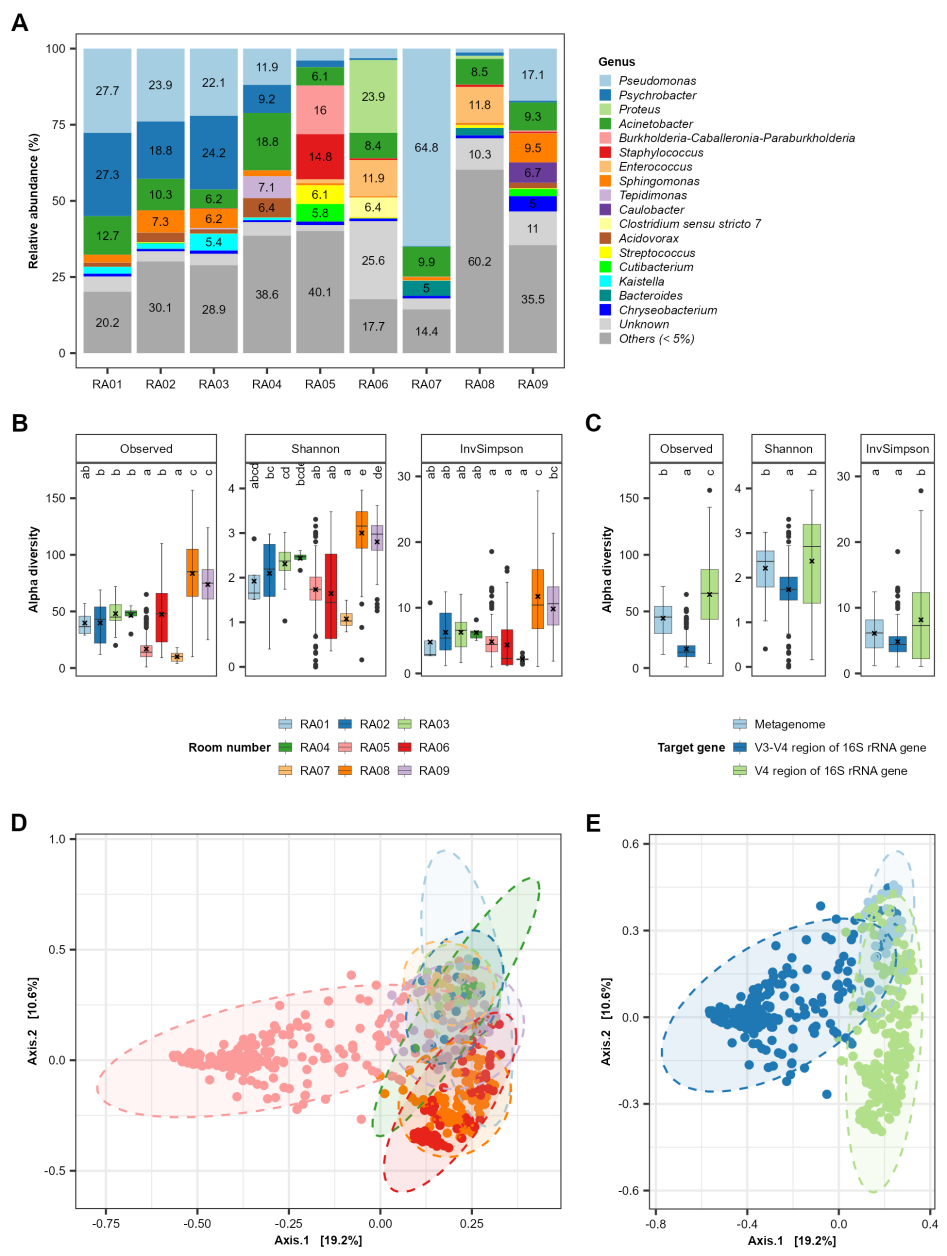
Alpha and beta diversity of persistent bacterial genera in meat processing plants. **(A)** The relative abundance of bacterial genera in the combined samples in each functional room. The room function is shown in Table 1. Panels **(B,C)** show the comparison of alpha diversity among different functional rooms and sequencing methods, respectively. The sequencing methods includes sequencing of metagenome DNA, V4 region of 16S rRNA gene DNA and V3-V4 region of 16S rRNA. Panels **(D,E)** show the PCoA plot distinguished with room numbers and sequencing methods, respectively.

A total of 17 genera each accounted for >5% of the microbiota in at least one of nine functional rooms (Figure 1A). On average, *Pseudomonas* (19.5%), *Acinetobacter* (10%), *Psychrobacter* (9.3%), *Sphingomonas* (3.3%), *Enterococcus* (2.8%), *Proteus* (2.8%), *Staphylococcus* (1.9%), *Burkholderia-Caballeronia-Paraburkholderia* (1.8%), *Acidovorax* (1.6%), and *Brevundimonas* (1.5%) were the top 10 most predominant genera in all functional rooms included (Supplementary Table S4). Rooms RA01-04 were at a pork cutting plant in Spain (Cobo-Díaz et al., 2021). No significant (*p* > 0.05) difference in either alpha (genus richness) or beta diversity (genus composition) was observed for these rooms (Figures 1B,D). However, variations in both alpha diversity and beta diversity were observed among the rooms from different plants (Figures 1B,D). The beef slaughter room (RA08) in an Australian plant and the pork cutting room (RA09) in a Canadian plant showed relative larger alpha diversity compared to other rooms (Figure 1B). In terms of beta diversity, all the rooms from different plants showed significant difference. Sequencing methods seem to affect the beta diversity, which may have contributed to the further distance between rooms RA01-04/RA05 and others (Figures 1D,E). RA01-04, RA05, and RA06-09 were investigated by sequencing metagenome DNA, amplicon of V3 region of 16S rRNA and amplicon of V4 region of 16S rRNA gene DNA in collected samples, respectively. Sequencing of cDNA of 16S rRNA focuses on the live bacteria in a sample, while direct DNA sequencing was not able to distinguish between live and dead bacterial cells. This also supports the observation in this review that RNA based method showed lower alpha diversity compared to DNA based methods (Figure 1C).

### 4.3 Potential correlation between persistent bacterial genera and a plant/function room

**Figure 2:**
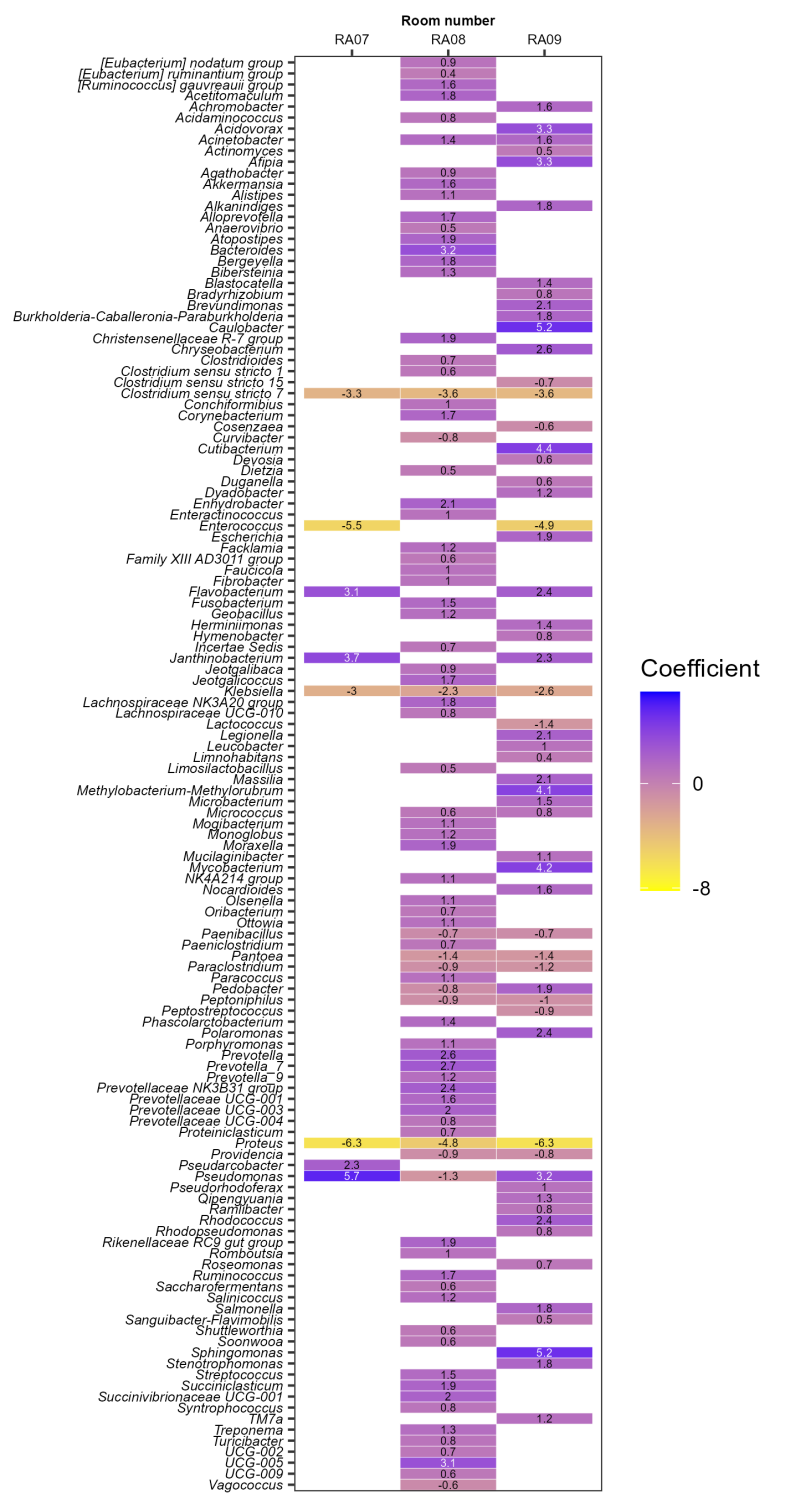
The association of persistent bacterial genera with each functional room investigated by sequencing of V4 region of 16S rRNA gene DNA. Room RA06 was used as a reference.

To investigate whether a bacterial genus has significant correlation with a specific plant/functional room, we performed correlation analysis using a R package MaAsLin2 (Mallick et al., 2021). The rooms (RA06-RA09) for which the same sequencing method was used, i.e., sequencing of V4 region of 16S rRNA gene, were included for comparison. RA06 (beef cutting room in an Australian meat plant) was used as a reference. A total of 127 bacterial genera each showed a significant (Benjamini-Hochberg corrected *p*-value < 0.001) positive or negative correlation with at least one functional room/plant (Figure 2). *Clostridium sensu stricto* 7, *Klebsiella*, *Proteus* were more abundant in RA06, or negatively associated with RA07-09 with correlation coefficient (effect estimate by MaAsLin2) ≤ −3.3, ≤ −2.3, and ≤−4.8, respectively (Figure 2). RA07 (beef cutting room) and RA09 (pork cutting room) were both located in Canada, the microbiota of which were more predominated with *Pseudomonas*, *Janthinobacterium*, and *Flavobacterium* but less predominated with *Enterococcus*, compared to other functional rooms. *Bacteroides* and UCG-005 were only positively associated with the beef slaughter room (RA08) in the Australia plant with coefficients >3. In addition, *Caulobacter, Sphingomonas, Cutibacerium, Mycobacterium, Methylobacterium-Methylorubrum, Acidovorax*, and *Afipia* were only positively correlated with RA09 with coefficients >3, the pork cutting room in a Canadian plant. The correlation between a specific genus with a meat plant found in this review may be attributable to several factors including different production practices, geographical regions and meat processing stages/functions of/in the included meat plants. Our previous study has shown meat products produced at different meat plants are predominated with different strains of *Carnobacterium*, a bacterial genus associated with the storage life of vacuum-packaged meat (Zhang et al., 2018). Whole genome sequencing data showed the meat production environment was the likely source of the contaminating *Carnobacterium* strains (Zhang et al., 2018). A better understanding of the persistent bacterial genera/species/strain in a meat plant will likely help to better control/predict the shelf life of produced meat products.

### 4.4 Bacterial co-existence network

We investigated the correlation between the relative abundance of bacterial genera, to explore the potential bacterial co-existence network in meat plant environments. A positive correlation indicates the possible co-existence between two bacterial genera, which may be attributed to their interaction in a micro-environment/biofilm, the preference for the same growth condition, or the same contamination pattern. For this purpose, we only included the functional rooms with ≥10 samples, i.e., RA02-03, RA05-06, and RA08-09. We first rarefied the sequencing data to 1,000 reads for each sample and then calculated the relative abundance of bacterial genera. Therefore, the co-existence network analysis in this review only focused on the bacterial genera with a relative abundance ≥0.1% in each functional room. Theoretically, the bacterial genus which accounts for <0.1% of the microbiota will get a zero as its relative abundance after data rarefaction. For statistical analysis, 0.01% was arbitrarily assigned to those zeros. A log transformation with 10 as the base was then performed for the percentage values. Therefore, the log values for relative abundance of <0.1, 0.1, 1, 10, and 100% were − 2, −1, 0, 1, and 2, respectively. Spearman’s rank correlation was performed for each combination of two genera for each functional room. The correlation with a *p* value <0.001 and coefficient > 0.5 or <−0.5 was regarded as significant. We looked at the combinations with significant correlation found in more than one functional room.

**Figure 3:**
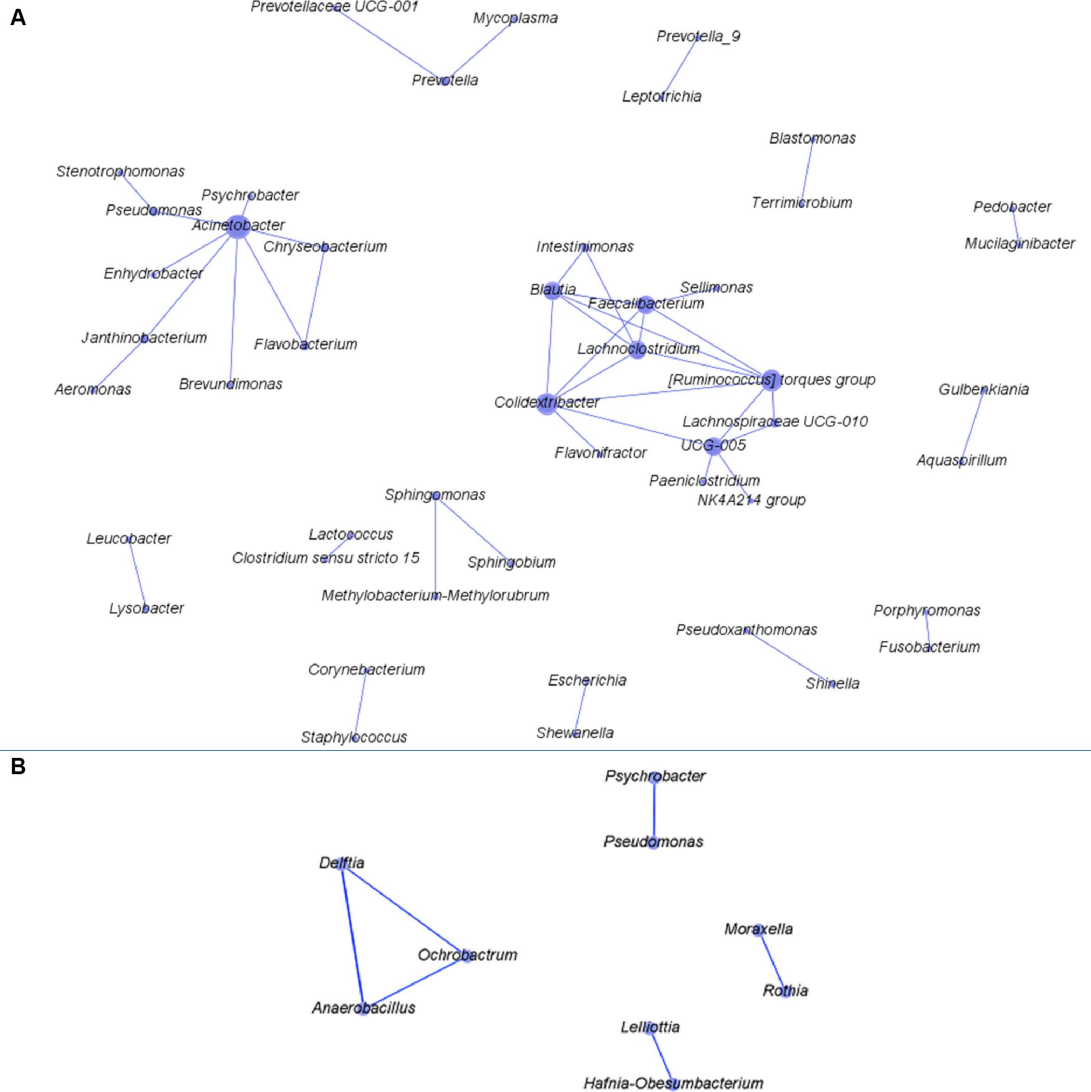
The network constructed based on the correlations between two bacterial genera found in more than two of the functional rooms with >10 samples. Panels **(A,B)** are networks constructed for bacteria from after-cleaning and before-cleaning samples.

Three and 42 combinations of bacterial genera were found to have significant correlation in three and two functional rooms, respectively (Figure 3A; Supplementary Figure S1). No significant correlations were found in >3 functional rooms. All the correlations were found positive except for *Escherichia* and *Psychrobacter* which were negatively correlated with a coefficient of −0.58 in RA09 (the pork cutting room in a Canadian plant; Supplementary Figure S1). However, the two genera had positive correlation in RA06 (a beef cutting room in an Australian plant). The significant correlations found in three functional rooms were between *Acinetobacter* and *Psychrobacter*, *Chryseobacterium* and *Flavobacterium*, and *Sphingomonas* and *Methylobacterium-Methylorubrum* (Supplementary Figure S1). A network based on the correlations was constructed (Figure 3A), which showed that *Acinetobacter* correlated with the greatest number of genera (*n* = 5) followed by *Psychrobacter* (*n* = 4), *Chryseobacterium* (*n* = 4), *Pedobacter* (*n* = 3), and *Prevotella* (*n* = 3).

### 4.5 Bacteria in meat processing environment before cleaning or during production

The meat plant samples in some studies included those collected before the routine cleaning or during meat production. We also analyzed the bacteria in these samples (*n* = 439), which were from conveyor belts, equipment surfaces, knives, aprons, gloves, etc. (Supplementary Table S3) in plants in Austria, USA, Italy, and Canada (Yang et al., 2016; Botta et al., 2020; Zwirzitz et al., 2020, 2021; Shedleur-Bourguignon et al., 2023; Supplementary Table S2). Like after-cleaning samples, three bacterial genera including *Pseudomonas* (9.7%), *Psychrobacter* (9.6%), and *Acinetobacter* (6.2%) also had average relative abundance of >5% in before-cleaning samples (Supplementary Table S4). *Delftia* was not predominant (≤0.6%) in any of functional rooms after cleaning but had relative abundance > 10% in two functional rooms before cleaning, including the pig slaughter room (RB01, 16.7%) and pork cutting room (RB02, 15.7%) in a meat plant in Austria (Supplementary Table S4). As the before- and after-cleaning samples were sequenced using different methods and were from different functional rooms in different geographical regions, we did not perform statistical comparison.

In four functional rooms with ≥10 samples, we found one (*Anaerobacillus* vs. *Delftia*) and five (*Rothia* vs. *Moraxella*; *Lelliottia* vs. *Hafnia-Obesumbacterium*; *Anaerobacillus* vs. *Ochrobactrum*; *Delftia* vs. *Ochrobactrum*; *Pseudomonas* vs. *Psychrobacter*) combinations of bacterial genera that were positively correlated in three and two functional rooms, respectively (Supplementary Figure S2). Among the six combinations, *Delftia, Anaerobacillus*, and *Ochrobactrum* all had significant correlation with two genera and others with one genus (Figure 3B). Studies on the co-existence network among meat plant microbiota are very scarce. Botta et al. recently explored the co-occurrence network for bacteria both on beef carcass and in slaughterhouse environments (Botta et al., 2022, 2023). The mechanisms mediating the pairwise co-existence/occurrence in both studies and those in our analysis are not clear. However, further relevant studies may help us better understand the micro-ecology of bacteria in meat production chain. Considering the co-existence network constructed for both before- and after-cleaning samples together, the bacterial genera with more connections in the network tended to have larger relative abundance than others. This finding suggests the co-existence relationship between predominant bacteria is easier to capture than their scarce counterparts. The contamination level of pathogens in meat processing plants is normally very low. Nevertheless, the information on its synergistic or antagonistic interaction with commensal bacteria in meat plant would be meaningful to food safety. For this purpose, a culture-dependent method is necessary.

## 5 Biofilm formation, an important mechanism by which bacteria persist in meat processing facilities

In meat processing environments, the survival of bacteria could be mediated by one or more of the following ways: inherent/increased resistance to biocides, shielded by surfaces (e.g., hard to reach spots) or meat debris, and protected by biofilms. Generic *E. coli* has long been used as an indicator to evaluate the hygienic condition in meat processing plant, the sensitivity of which to biocides have been reported by various researchers. Aarestrup and Hasman (2004) compared the susceptibility of 202 *E. coli* isolates from Danish cattle, broilers, and pigs to a number of biocides including quaternary compounds (QAC), the most commonly used sanitizer in food settings. Most *E. coli* had minimum inhibitory concentration (MIC) values of 64 ppm and the highest MIC observed for *E. coli* was 128 ppm. The mean MIC values for QAC and sodium hypochlorite of both persisting (n = 50, genotype recovered more than twice) and transient (n = 50, genotype recovered once) *E. coli* strains collected from fabrication equipment at a beef packing plant did not differ significantly and were well below the in-use concentration for both sanitizers (QAC, 200 ppm; sodium hypochlorite, 200 ppm active chlorine; Yang et al., 2018). Lavilla Lerma et al. (2015) tested the sensitivity of pseudomonads isolated from a goat and lamb slaughterhouse to biocides including triclosan, cetrimide, benzalkonium chloride. They found the included *Pseudomonas* isolates were all highly susceptible to industry formulations of these biocides. There is very limited information on the resistance of other bacterial species to the sanitizers often used in meat plants. However, the available studies suggest the likelihood of biocide resistance of planktonic cells of meat plant bacteria being the main mechanism of persistence in meat processing environment would be low.

### 5.1 Biofilm structure and bacterial survival

The inability to remove biofilms poses a risk for ongoing microbial contamination in meat processing facilities. Biofilms are complex structures with bacterial cells embedded in extracellular polymeric substances (EPS) also known as the glycocalyx, and are widely acknowledged as the dominant mode of microorganism existence (Watnick and Kolter, 2000; White et al., 2011). The EPS is a mixture of polysaccharides, proteins, lipids, and highly hydrated nucleic acids that acts as a shield and source of water, protecting the microorganisms within it against desiccation, biocides, and other environmental stressors (Coughlan et al., 2016; Yin et al., 2019). EPS also aids in the retention and concentration of essential nutrients (White et al., 2011). Sessile cells exhibit altered physiological characteristics compared to their planktonic counterparts, including changes in gene expression, metabolism, and resistance to antimicrobial agents (Costerton et al., 1999). Thus, the biofilm mode of living provides a better fitness to bacterial survival through the physical barrier provided by EPS, efflux systems, differentiation of bacterial cells into a dormant state, and the modification of the micro-environment. This can render a particular sanitizer less effective (Giaouris et al., 2014), which is certainly relevant in meat processing environments. The persistent *E. coli* population in the study by Yang et al. (2018) had a large fraction of biofilm formers when tested at 15°C for 6 days. Wang et al. (2016) also reported strong biofilm forming ability and higher tolerance to sanitizers in biofilms of “high-event period” *E. coli* O157:H7 strains.

The attachment and subsequent biofilm formation of bacteria depends on interactions between bacteria and the environment (Garrett et al., 2008). During meat processing operations, the wetting of processing surfaces such as conveyor belts by meat juice and the adsorption of food residues to surfaces provide a conditioning layer which modifies surface properties favorably for bacterial attachment and subsequent growth (Yin et al., 2019; Carrascosa et al., 2021). In addition, biofilms formed initially under high humidity conditions can dehydrate, resulting in prolonged bacterial survival (Adator et al., 2018; Nan et al., 2022).

### 5.2 Biofilm forming ability of meat production related bacteria

Numerous studies have investigated the biofilm forming ability of *E. coli*. A larger proportion of generic *E. coli* tend to be biofilm formers compared to pathogenic *E. coli*. Uhlich et al. (2014) examined the biofilm formation of clinical *E. coli* O157 strains on polystyrene surface under various conditions and found that most (49/54) strains did not form any measurable biofilms. Of the five biofilm formers, only one was considered to be strong. Similarly, Wang et al. (2012) reported that two out of 30 STEC strains from various sources were biofilm formers when cultured on polystyrene surface for 24 h. Generic *E. coli* strains (n = 700) collected from carcasses along the dressing process and during chilling, from meat products and fabrication equipment surfaces were compared with Top 7 *E. coli* strains (*n* = 745) recovered from cattle in their biofilm formation under equivalent conditions (Stanford et al., 2021). Biofilm formers accounted for 7.1% of the total Top 7 strains and 42.9% of the total generic *E. coli* population. Comparative genomic analysis of Top 7 *E. coli* strains revealed that more virulence factors were associated with the non-biofilm forming population and acid resistant population, while they were least present in populations where heat resistance genes and metal resistance genes were enriched (Fang et al., 2023). These findings suggest that there is a divergence between environmental fitness and virulence of *E. coli*. A number of factors could have driven this divergence: 1. The much lower temperature in the meat fabrication environment (mostly ≤ 10°C during operation and ≤ 15°C during downtime) compared to the host’s intestines (35°C); 2. The potential encounter of physical (desiccation from equipment drying) and chemical (sanitizers/cleaners) bactericides.

Much less attention has been drawn to spoilage or resident microbiota in meat processing plants. Among the most predominant persistent bacterial genera revealed by our meta-analysis on meat plant microbiota, *Pseudomonas* strains have been proven to be biofilm-formers under chilled conditions simulating meat production environment (Morimatsu et al., 2012; Liu et al., 2013; Visvalingam et al., 2019; Wickramasinghe et al., 2020; Wagner et al., 2021). *Acinetobacter* can be a strong or weak biofilm-former depending on the species/strain identity of the isolates (Liu et al., 2013; Visvalingam et al., 2019). Yang et al. (2023b) reported the predominance of *Pseudomonas* and *Acinetobacter* isolates in biofilms formed by meat plant microbiota on stainless steel coupons at 15°C. Both species also predominated in the multi-species biofilms which formed on conveyor belts under conditions simulating meat processing environments and retained its stability even when conveyor belts were rinsed by QAC, peracetic acid or H_2_O (Fagerlund et al., 2017). *Psychrobacter* does not seem to be a strong biofilm-former although it often predominates in meat packing plants. A *Psychrobacter* strain did not form measurable biofilms quantified using crystal violet staining (Visvalingam et al., 2019). Wagner et al. (2021) reported the lowest bacterial load of the tested *Psychrobacter* strains on stainless steel slides compared to other meat plant microbiota (e.g., *Pseudomonas fragi*, *Acinetobacter harbinensis*, *Microbacterium* sp., *Carnobacterium maltaromaticum*, etc.). Factors such as the initial load from animal intestines and their ability to grow at low temperatures, rather than their ability to form biofilms, may have contributed to the predominance and persistence of *Psychrobacter* in meat processing environments. *Sphingomonas* is a group of strictly aerobic Gram-negative bacteria and ranked among the top four most predominant genus in our meta-analysis. It was a moderate biofilm former in the study by Liu et al. (2013) and did not form measurable biofilms in the study by Visvalingam et al. (2019), respectively. Castaño-Arriba et al. (2020) tested 200 *Enterococcus* spp. recovered from red meat and poultry products, and found they all produced weak, moderate or strong biofilms on polystyrene microwell plates depending on the isolates. Very little information has been found for other bacterial species reported in meat plant microbiota.

### 5.3 The effects of background microbiota on biofilm formation by *Escherichia coli*

The diverse species of bacteria found on/in processing equipment/environments may affect biofilm formation by *E. coli* (Møretrø et al., 2013; Wang et al., 2018; Fagerlund et al., 2021). When co-cultured with bacteria recovered from processing environments in dual-species cultures, STEC strains showed interactions in a STEC-strain and companion-strain dependent manner, with both synergistic and antagonistic effects being observed (Fang et al., 2022; Nan et al., 2022). For example, biofilms formed by *Pseudomonas aeruginosa* were found to be antagonistic against STEC O103 strains (Nan et al., 2022). The Gram-positive bacterium *Microbacterium phyllosphaerae* was synergistic for biofilm formation with various STEC strains in dual-species cultures (Fang et al., 2022). As such, the presence of background bacteria must be considered when evaluating the biofilm formation of *E. coli*. A recent study reported that a STEC O157:H7 strain was able to co-develop biofilms with post-sanitation process equipment surface microbiota and insert into biofilms developed by such microbial communities, but could not form biofilms on its own when evaluated under the same incubation conditions (Yang et al., 2023b). This particular O157:H7 strain also lacks the ability to produce curli or cellulose at the temperature (15°C) used for biofilm formation. A similar phenomenon was observed for generic *E. coli* strain, PHL565, which was not able to adhere to a glass surface on its own, but it could do so when co-cultured with *P. putida* MT2 (Castonguay et al., 2006). The survival of STEC strains could be enhanced by background microbiota through multispecies biofilms in meat plants. The importance of effective cleaning and sanitation of fabrication environments cannot be overstated. Targeting the commensal background microbiota in meat processing environment rather than individual pathogen strains based on their biofilm forming ability may be a more rewarding approach, from a pathogen control standpoint. In addition, studies on commensal bacteria in meat plants have been mainly on genus level and the species identity of recovered isolates is largely unknown let alone the strain level identification. Phenotypic characterization of these background bacteria combining genomic analysis will help with the development of effective biofilm control/removal measures.

### 5.4 Genomic analysis on biofilm formation related genes with a focus on *Escherichia coli*

The development of biofilms can be divided into five discrete steps: initial reversible attachment of planktonic cells to a surface; irreversible attachment; microcolony growth; maturation (macrocolony); and dissolution (dispersal) which releases bacterial cells back into planktonic state and a new cycle may start (Van Houdt and Michiels, 2005). Adhesins and fimbriae are involved in the attachment of bacteria to the surfaces, allowing bacterial cells to form microcolonies (White et al., 2011). For *E. coli*, the fimbrial adhesin curli has been reported to be essential for biofilm formation (Uhlich et al., 2013). In addition to adhesins, cellulose, flagella, poly-β-1,6-N-acetyl-D-glucosamine (PGA), and colanic acid could also be involved in *E. coli* biofilm formation at different stages (Beloin et al., 2008). Consequently, biofilm formation is an orchestrated process of the work of many genes including those encoding for attachment apparatus, EPS, and their respective regulatory genes, as well as global regulatory genes for quorum sensing and the stationary phase sigma factor. Secretion systems have also been reported to play a crucial role in biofilm formation by facilitating the transport of various molecules, including proteins and polysaccharides, across the bacterial cell envelope and into the extracellular matrix (O’Toole and Kolter, 1998; Costa et al., 2015; Römling and Galperin, 2015).

*Escherichia coli* O157:H7 is the top ranked pathogenic STEC serotype associated with human outbreaks. To date, there has been no published literature inquiring into genomic features related to biofilm formation of *E. coli* O157:H7 on a population perspective. We hence scrutinized the presence of biofilm formation related genes in a number of O157:H7 strains for which both the genomes and origin information were available. A total of 98 genomes of O157:H7 strains were included, which were derived from cattle (*n* = 25), clinical samples/humans (*n* = 43), package lettuce (*n* = 1) beef products (*n* = 11). Eighteen strains of unknown origin were also included. Those strains were provided by the Canadian Food Inspection Agency (CFIA), the Food and Drug Administration (FDA) or the United State Department of Agriculture (USDA), and thus the likely origin would be food or animals. These strains originated from USA, UK, Japan, China, Mexico, Argentina, Canada, Germany, Denmark, and Brazil. We also included two non-STEC *E. coli* strains as references, *E. coli* K12 substr. MG1655 and ATCC 11775 (O1:K1:H7). Strain MG1655 is non-pathogenic and closely resembles wild-type *E. coli*, while ATCC 11775 was isolated from a patient with urinary infection and positive for cellulose and fimbria production. We utilized a comparative systems approach to analyze protein families across selected genomes. The genome annotation was carried out using RASTtk4, focusing on intra-genus comparisons (PLfams; Overbeek et al., 2005, 2014).

**Figure 4:**
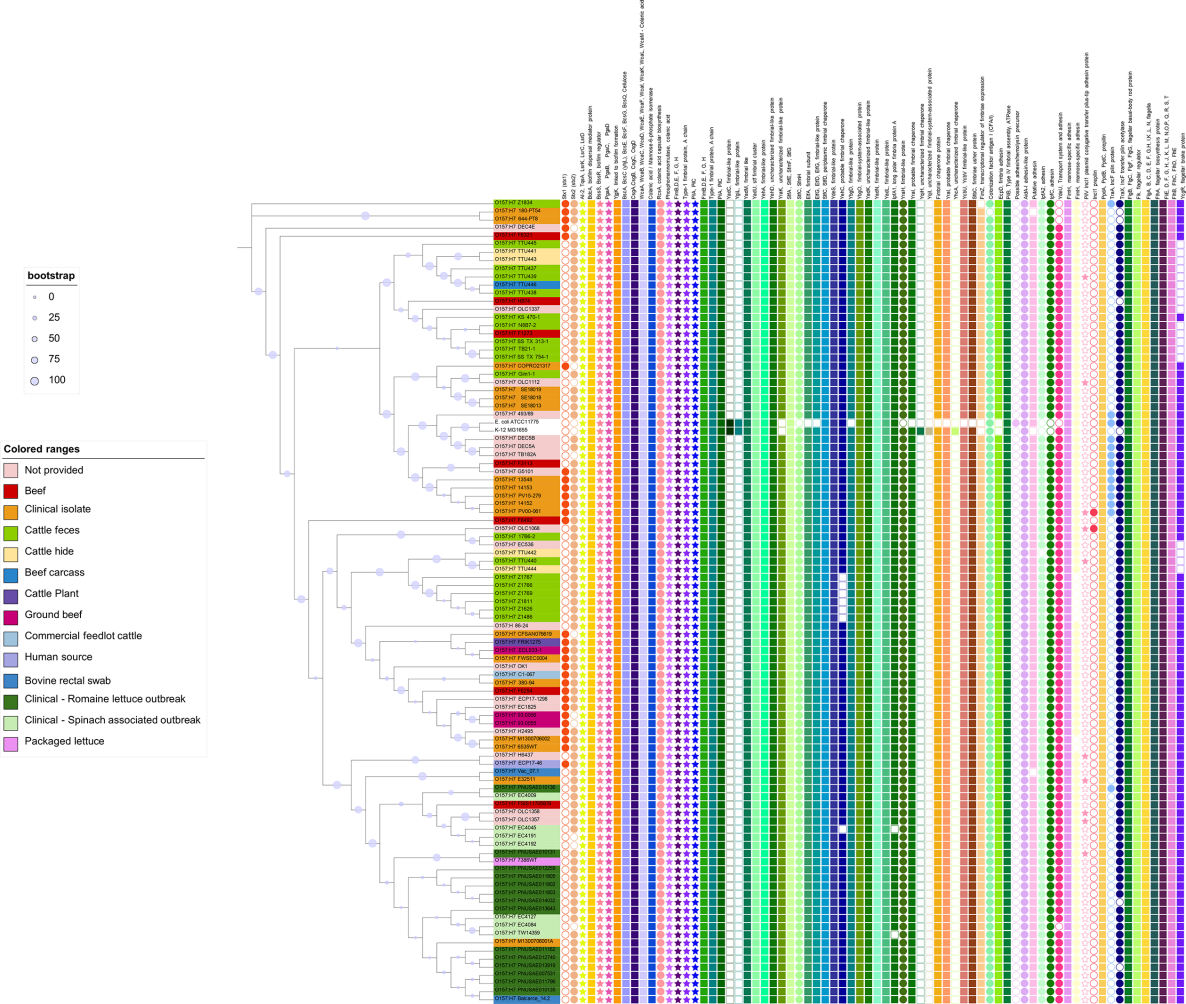
Biofilm related genes in *Escherichia coli* O157:H7 strains. The phylogenetic tree was constructed using the core genomes of the included genomes. *Escherichia coli* K12 and ATCC 11775 were included as references. The encoded gene products of relevant genes are annotated on the top of the figure. Solid-colored shapes represent the presence of relevant genes, while non-colored filled shapes indicate absence.

A total of 150 biofilm formation related genes were examined in these genomes, as listed in Figure 4. Associated proteins were grouped together in the figure, such as FlhB, FlhC, FlhD, and FlhD. Interestingly, the majority of those genes were found in all the genomes regardless of their isolation source. These included genes encoding proteins associated with quorum sensing (TqsA, LuxS, and TnaA), dedicated to biofilm regulation (BssS, BssR, and TabA), and related to biosynthesis and regulation of EPS matrix (PgaABCD, BcsEFGQ), curli (CsgABCD), and colanic acid (WcaABCDFIKLM, RcsA; Barnhart and Chapman, 2006; Domka et al., 2006; Itoh et al., 2008; Kim et al., 2009; Zhang and Poh, 2018). The genes encoding bacterial appendages, such as type-1 fimbriae (FimBEFGH) and type-4 fimbriae (pili, PilABCMNOPQT), and flagella (FlgA-N, FlgJ, Flk, FlhA) were found in all the genomes as well (O’Toole et al., 2000; Paranjpye and Strom, 2005; Charbonneau and Mourez, 2007; Kim et al., 2009; Belas, 2014). It has been hypothesized that 95% of O157:H7 isolates are non-biofilm-formers due to an insertion in the gene encoding a transcription factor (MlrA) and variation in a gene encoding a sigma regulator (RpoS), which limit curli expression and biofilm formation in O157:H7 (Uhlich et al., 2013). However, other research has shown that STEC that does not produce curli or cellulose phenotypically are able to form biofilms (Adator et al., 2018; Nan et al., 2022). The biofilm forming ability of the O157:H7 strains in this review needs further study. Nevertheless, the presence of these biofilm related genes in all O157:H7 strains underscores their fundamental importance in the ecological success of a diverse group of O157:H7 strains.

Plasmids have been reported to carry accessory genes influencing biofilm formation, particularly those related to surface attachment (Gama et al., 2020). Our analysis identified gene encoding proteins associated with conjugative plasmids in some selected genomes. Specifically, *pilV* (encoding IncI1 plasmid conjugative transfer pilus-tip adhesin protein), *pilS* (IncI1 plasmid conjugative transfer prepilin), *traA* (IncF plasmid conjugative transfer pilin protein), and *traX* (IncF plasmid conjugative transfer pilin acetylase) were more prevalent in O157:H7 and ATCC11775 strains. Notably, no plasmid-related proteins were found in *E. coli* K12.

**Figure 5:**
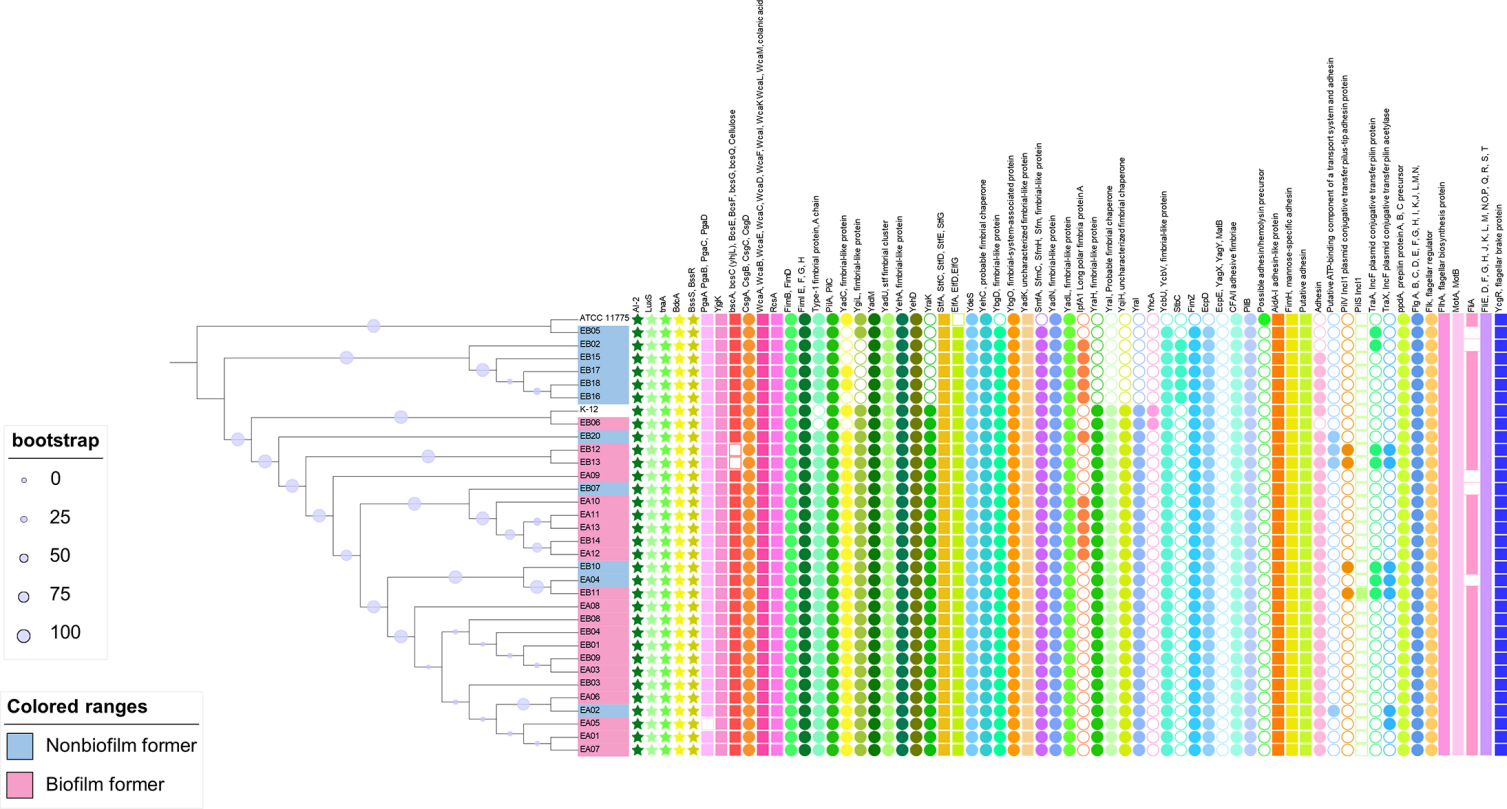
Biofilm related genes in genetic *Escherichia coli* strains recovered from meat cutting plants. The phylogenetic tree was constructed using the core genomes of the included genomes. *Escherichia coli* K12 and ATCC 11775 were included as references. The encoded gene products of relevant genes are annotated on the top of the figure. Solid-colored shapes represent the presence of relevant genes, while non-colored filled shapes indicate absence.

Simply comparing the O157:H7 strains to two generic *E. coli* strains may not provide a comprehensive understanding of the underlying patterns. Consequently, we conducted a second comparative analysis that involved a broader spectrum of generic *E. coli* strains. This expanded analysis encompassed 32 isolates of generic *E. coli* obtained from meat processing equipment (Yang et al., 2023a). Our analysis revealed the consistent presence of genes associated with biofilm formation across all the generic *E. coli* genomes examined (Figure 5). Furthermore, it is noteworthy that generic *E. coli* strains originating from beef processing facilities exhibited a shared genetic repertoire related to biofilm formation, mirroring similar patterns observed in the O157:H7 strains. However, differences emerged in genes associated with adhesins, fimbria-like proteins, as well as the presence of adhesins or pili carried by plasmids.

Information regarding biofilm formation phenotype of meat plant *E. coli* at 15°C was available and highlighted in Figure 5. However, no clear patterns were observed regarding the association between the absence of presence of relevant genes and their ability to form biofilms at 15°C. Neither group showed overrepresentation of genes according to the original genomic analysis performed by Yang et al. (2023a). It is important to acknowledge that the ability to form biofilms at 15°C, as depicted in our data, may not be indicative of the strains’ complete phenotypic biofilm-forming capabilities, given the variability in environmental conditions within meat processing facilities. Further research is needed to explore the full spectrum of biofilm formation in these strains under different conditions.

Relevant studies have shown the presence of more functional genes related to fimbria, flagella, curli, cellulose production, adhesins in *E. coli* could improve biofilm formation (Niba et al., 2007; Wang, 2019). On the other hand, an engineered *E. coli* strain with 17.6% of the parental genome removed including the genes involved in the synthesis of various cell structures such as type I fimbriae, curli, EPS, and the quorum sensing molecule autoinducer-2 (AI-2) is able to develop mature biofilms (May and Okabe, 2011). A study of pellicle (air-liquid biofilms) and non-pellicle forming *E. coli* found differences in the regulatory region of curli biosynthesis, but not in the absence or presence of genes in the two groups (Xu et al., 2023). Collectively, these findings suggest that three aspects could all be relevant for biofilm formation in *E. coli*: genetic determinants of cellular apparatus involved in biofilm formation, overall genetic background of individual strains, and variations in regulatory genes.

## 6 Conclusion

The process of converting livestock to meat products is very complex. Throughout the whole process, many factors can affect the microbial ecology in meat facilities and the final meat products. The antimicrobial intervention type, chemical concentration, spraying method, and temperature of the antimicrobial strategies applied in meat plants may have different decontamination effects and lead to different compositions of bacteria on the applied surfaces. For example, *Psychrobacter* and *Pseudomonas* predominated among bacterial population on spray chilled and dry chilled carcasses, respectively (Yang et al., 2017b). In fact, the collective incoming microbiota, and any condition that differentially impacts members of the microbial communities along the entire process may have consequences on transient and residential microbiota.

The bacteria residing on meat fabrication equipment and other environmental surfaces have been regarded as an important source contaminating meat products. Diverse bacteria can persist in meat plants, among which *Pseudomonas*, *Acinetobacter*, *Psychrobacter, Sphingomonas, Enterococcus, Proteus, Staphylococcus, Burkholderia-Caballeronia-Paraburkholderia, Acidovorax*, and *Brevundimonas* are the top 10 most predominant genera. However, information on the predominant bacteria identified at species/strains level is largely lacking. Biofilm is believed to be an important mechanism through which bacteria persist in meat plants. The commensal/residential bacteria may have synergistic or antagonistic effects for pathogenic bacteria such as STEC to form biofilm. They may also enhance the biofilm formation of pathogens who otherwise do not form biofilms on their own. Targeted cleaning and sanitizing efforts against residential microbiota may be rewarding in both safety and storage stability of meat products.

## Author contributions

XY: Conceptualization, Funding acquisition, Resources, Supervision, Writing – original draft, Writing – review & editing. CN -B: Data curation, Formal Analysis, Funding acquisition, Investigation, Methodology, Resources, Writing – original draft, Writing – review & editing. PZ: Data curation, Formal Analysis, Funding acquisition, Investigation, Methodology, Resources, Writing – original draft, Writing – review & editing.

## Footnotes

^1^ https://www.ncbi.nlm.nih.gov/

^2^ https://github.com/ncbi/sra-tools

^3^ https://github.com/smdabdoub/kraken-biom

^4^ https://github.com/vegandevs/vegan

## References

[ref1] Aarestrup F. M. Hasman H. (2004). Susceptibility of different bacterial species isolated from food animals to copper sulphate, zinc chloride and antimicrobial substances used for disinfection. Vet. Microbiol. 100, 83–89. doi: 10.1016/j.vetmic.2004.01.013, PMID: 15135516

[ref2] Adator E. H. Cheng M. Holley R. Mcallister T. Narvaez-Bravo C. (2018). Ability of Shiga toxigenic *Escherichia coli* to survive within dry-surface biofilms and transfer to fresh lettuce. Int. J. Food Microbiol. 269, 52–59. doi: 10.1016/j.ijfoodmicro.2018.01.014, PMID: 29421358

[ref3] Algino R. J. Ingham S. C. Zhu J. (2007). Survey of antimicrobial effects of beef carcass intervention treatments in very small state-inspected slaughter plants. J. Food Sci. 72, M173–M179. doi: 10.1111/j.1750-3841.2007.00386.x, PMID: 17995740

[ref4] Arthur T. M. Bono J. L. Kalchayanand N. (2014). Characterization of *Escherichia coli* O157:H7 strains from contaminated raw beef trim during “high event periods”. Appl. Environ. Microbiol. 80, 506–514. doi: 10.1128/AEM.03192-13, PMID: 24212567 PMC3911080

[ref5] Arthur T. M. Bosilevac J. M. Nou X. Shackelford S. D. Wheeler T. L. Kent M. P.. (2004). *Escherichia coli* O157 prevalence and enumeration of aerobic bacteria, Enterobacteriaceae, and *Escherichia coli* O157 at various steps in commercial beef processing plants. J. Food Prot. 67, 658–665. doi: 10.4315/0362-028X-67.4.658 15083715

[ref6] Barnhart M. M. Chapman M. R. (2006). Curli biogenesis and function. Annu. Rev. Microbiol. 60, 131–147. doi: 10.1146/annurev.micro.60.080805.142106, PMID: 16704339 PMC2838481

[ref7] Belas R. (2014). Biofilms, flagella, and mechanosensing of surfaces by bacteria. Trends Microbiol. 22, 517–527. doi: 10.1016/j.tim.2014.05.002, PMID: 24894628

[ref8] Beloin C. Roux A. Ghigo J. M. (2008). *Escherichia coli* biofilms. Curr. Top. Microbiol. Immunol. 322, 249–289. doi: 10.1007/978-3-540-75418-3_12, PMID: 18453280 PMC2864707

[ref9] Bessegatto J. A. Paulino L. R. Lisboa J. A. N. Alfieri A. A. Montemor C. H. Medeiros L. P.. (2017). Changes in the fecal microbiota of beef cattle caused by change in management and the use of virginiamycin as a growth promoter. Res. Vet. Sci. 114, 355–362. doi: 10.1016/j.rvsc.2017.06.011, PMID: 28675873

[ref10] Blagojevic B. Antic D. Ducic M. Buncic S. (2012). Visual cleanliness scores of cattle at slaughter and microbial loads on the hides and the carcases. Vet. Rec. 170:563. doi: 10.1136/vr.100477 22505244

[ref11] Botta C. Ferrocino I. Pessione A. Cocolin L. Rantsiou K. (2020). Spatiotemporal distribution of the environmental microbiota in food processing plants as impacted by cleaning and sanitizing procedures: the case of slaughterhouses and gaseous ozone. Appl. Environ. Microbiol. 86, e01861–e01820. doi: 10.1128/AEM.01861-20 32978124 PMC7657643

[ref12] Botta C. Franciosa I. Alessandria V. Cardenia V. Cocolin L. Ferrocino I. (2022). Metataxonomic signature of beef burger perishability depends on the meat origin prior grinding. Food Res. Int. 156:111103. doi: 10.1016/j.foodres.2022.111103 35650996

[ref13] Botta C. Franciosa I. Coisson J. D. Ferrocino I. Colasanto A. Arlorio M.. (2023). Beef carcass microbiota after slaughtering and primary cooling: a metataxonomic assessment to infer contamination drivers. Food Res. Int. 174:113466. doi: 10.1016/j.foodres.2023.113466 37986409

[ref14] Callahan B. J. Mcmurdie P. J. Rosen M. J. Han A. W. Johnson A. J. Holmes S. P. (2016). Dada2: high-resolution sample inference from Illumina amplicon data. Nat. Methods 13, 581–583. doi: 10.1038/nmeth.3869 27214047 PMC4927377

[ref15] Carrascosa C. Raheem D. Ramos F. Saraiva A. Raposo A. (2021). Microbial biofilms in the food industry-a comprehensive review. Int. J. Environ. Res. Public Health 18:14. doi: 10.3390/ijerph18042014, PMID: 33669645 PMC7922197

[ref16] Casas D. E. Vargas D. A. Randazzo E. Lynn D. Echeverry A. Brashears M. M.. (2021). In-plant validation of novel on-site ozone generation technology (bio-safe) compared to lactic acid beef carcasses and trim using natural microbiota and *Salmonella* and *E. coli* O157:H7 surrogate enumeration. Foods 10:1002. doi: 10.3390/foods10051002 34064320 PMC8147810

[ref17] Castaño-Arriba A. González-Machado C. Igrejas G. Poeta P. Alonso-Calleja C. Capita R. (2020). Antibiotic resistance and biofilm-forming ability in Enterococcal isolates from red meat and poultry preparations. Pathogens 9:1021. doi: 10.3390/pathogens9121021, PMID: 33287445 PMC7761845

[ref18] Castonguay M. H. Van Der Schaaf S. Koester W. Krooneman J. Van Der Meer W. Harmsen H.. (2006). Biofilm formation by *Escherichia coli* is stimulated by synergistic interactions and co-adhesion mechanisms with adherence-proficient bacteria. Res. Microbiol. 157, 471–478. doi: 10.1016/j.resmic.2005.10.003, PMID: 16376056

[ref19] CDC. (2014). *E. coli (Escherichia coli)*. Available at: https://www.cdc.gov/ecoli/general/index.html (Accessed 25 October 2023).

[ref20] CFIA (2010). “Ante and post-mortem procedures, dispositions, monitoring and controls—meat species, ostriches, rheas and emus” in Meat Hygiene Manual of Procedures, https://epe.lac-bac.gc.ca/100/206/301/cfia-acia/2011-09-21/inspection.gc.ca/english/fssa/meavia/man/mane.shtml

[ref21] Charbonneau M. E. Mourez M. (2007). Functional organization of the autotransporter adhesin involved in diffuse adherence. J. Bacteriol. 189, 9020–9029. doi: 10.1128/JB.01238-07, PMID: 17933890 PMC2168634

[ref22] Chen S. Zhou Y. Chen Y. Gu J. (2018). Fastp: an ultra-fast all-in-one Fastq preprocessor. Bioinformatics 34, i884–i890. doi: 10.1093/bioinformatics/bty560 30423086 PMC6129281

[ref23] Cherifi T. Arsenault J. Quessy S. Fravalo P. (2022). Co-occurrence of *L. monocytogenes* with other bacterial genera and bacterial diversity on cleaned conveyor surfaces in a swine slaughterhouse. Microorganisms 10:613. doi: 10.3390/microorganisms10030613, PMID: 35336188 PMC8948719

[ref24] Cobo-Díaz J. F. Alvarez-Molina A. Alexa E. A. Walsh C. J. Mencía-Ares O. Puente-Gómez P.. (2021). Microbial colonization and resistome dynamics in food processing environments of a newly opened pork cutting industry during 1.5 years of activity. Microbiome 9, 1–19. doi: 10.1186/s40168-021-01131-9 34645520 PMC8515711

[ref25] Costa T. R. D. Felisberto-Rodrigues C. Meir A. Prevost M. S. Redzej A. Trokter M.. (2015). Secretion systems in gram-negative bacteria: structural and mechanistic insights. Nat. Rev. Microbiol. 13, 343–359. doi: 10.1038/nrmicro3456, PMID: 25978706

[ref26] Costerton J. W. Stewart P. S. Greenberg E. P. (1999). Bacterial biofilms: a common cause of persistent infections. Science 284, 1318–1322. doi: 10.1126/science.284.5418.1318 10334980

[ref27] Coughlan L. M. Cotter P. D. Hill C. Alvarez-Ordóñez A. (2016). New weapons to fight old enemies: novel strategies for the (bio)control of bacterial niofilms in the food industry. Front. Microbiol. 7:1641. doi: 10.3389/fmicb.2016.01641 27803696 PMC5067414

[ref28] Domka J. Lee J. Wood T. K. (2006). YliH (BssR) and YceP (BssS) regulate *Escherichia coli* K-12 biofilm formation by influencing cell signaling. Appl. Environ. Microbiol. 72, 2449–2459. doi: 10.1128/AEM.72.4.2449-2459.2006, PMID: 16597943 PMC1448992

[ref29] Dowd S. E. Callaway T. R. Wolcott R. D. Sun Y. Mckeehan T. Hagevoort R. G.. (2008). Evaluation of the bacterial diversity in the feces of cattle using 16S rdna bacterial tag-encoded Flx amplicon pyrosequencing (btefap). BMC Microbiol. 8:125. doi: 10.1186/1471-2180-8-125, PMID: 18652685 PMC2515157

[ref30] Ekong P. S. Sanderson M. W. Cernicchiaro N. (2015). Prevalence and concentration of *Escherichia coli* O157 in different seasons and cattle types processed in North America: a systematic review and meta-analysis of published research. Prev. Vet. Med. 121, 74–85. doi: 10.1016/j.prevetmed.2015.06.019 26153554

[ref31] Essendoubi S. Yang X. King R. Keenliside J. Bahamon J. Diegel J.. (2020). Prevalence and characterization of *Escherichia coli* O157:H7 on pork carcasses and in swine colon contents from provincially licensed abattoirs in Alberta, Canada. J. Food Prot. 83, 1909–1917. doi: 10.4315/JFP-20-146, PMID: 32584991

[ref32] Fagerlund A. Langsrud S. Møretrø T. (2021). Microbial diversity and ecology of biofilms in food industry environments associated with *Listeria monocytogenes* persistence. Curr. Opin. Food Sci. 37, 171–178. doi: 10.1016/j.cofs.2020.10.015

[ref33] Fagerlund A. Møretrø T. Heir E. Briandet R. Langsrud S. (2017). Cleaning and disinfection of biofilms composed of *Listeria monocytogenes* and background microbiota from meat processing surfaces. Appl. Environ. Microbiol. 83:17. doi: 10.1128/AEM.01046-17, PMID: 28667108 PMC5561291

[ref34] Fang Y. Tran F. Stanford K. Yang X. (2023). Stress resistance and virulence gene profiles associated with phylogeny and phenotypes of *Escherichia coli* from cattle. J. Food Prot. 86:100122. doi: 10.1016/j.jfp.2023.100122, PMID: 37355007

[ref35] Fang Y. Visvalingam J. Zhang P. Yang X. (2022). Biofilm formation by non-O157 Shiga toxin-producing *Escherichia coli* in monocultures and co-cultures with meat processing surface bacteria. Food Microbiol. 102:103902. doi: 10.1016/j.fm.2021.103902 34809934

[ref36] Gama J. A. Fredheim E. G. A. Cléon F. Reis A. M. Zilhão R. Dionisio F. (2020). Dominance between plasmids determines the extent of biofilm formation. Front. Microbiol. 11:2070. doi: 10.3389/fmicb.2020.02070, PMID: 32983050 PMC7479130

[ref37] Garrett T. R. Bhakoo M. Zhang Z. (2008). Bacterial adhesion and biofilms on surfaces. Prog. Nat. Sci. 18, 1049–1056. doi: 10.1016/j.pnsc.2008.04.001

[ref38] Gaskins H. R. Collier C. T. Anderson D. B. (2002). Antibiotics as growth promotants: mode of action. Anim. Biotechnol. 13, 29–42. doi: 10.1081/ABIO-120005768 12212942

[ref39] Giaouris E. Heir E. Hébraud M. Chorianopoulos N. Langsrud S. Møretrø T.. (2014). Attachment and biofilm formation by foodborne bacteria in meat processing environments: causes, implications, role of bacterial interactions and control by alternative novel methods. Meat Sci. 97, 298–309. doi: 10.1016/j.meatsci.2013.05.023 23747091

[ref40] Gill C. O. (2005). “Haccp in the processing of fresh meat” in Improving the safety of fresh meat. ed. Sofos J. N. (Cambridge, U.K.: Crc/Woodhead Publishing Limited)

[ref41] Gill C. O. (2009). Effects on the microbiological condition of product of decontaminating treatments routinely applied to carcasses at beef packing plants. J. Food Prot. 72, 1790–1801. doi: 10.4315/0362-028X-72.8.1790 19722420

[ref42] Greig J. D. Waddell L. Wilhelm B. Wilkins W. Bucher O. Parker S.. (2012). The efficacy of interventions applied during primary processing on contamination of beef carcasses with *Escherichia coli*: a systematic review-meta-analysis of the published research. Food Control 27, 385–397. doi: 10.1016/j.foodcont.2012.03.019

[ref43] Haque M. Bosilevac J. M. Chaves B. D. (2022). A review of Shiga-toxin producing *Escherichia coli* (Stec) contamination in the raw pork production chain. Int. J. Food Microbiol. 377:109832. doi: 10.1016/j.ijfoodmicro.2022.109832 35834920

[ref44] Heiman K. E. Mody R. K. Johnson S. D. Griffin P. M. Gould L. H. (2015). *Escherichia coli* O157 outbreaks in the United States, 2003-2012. Emerg. Infect. Dis. 21, 1293–1301. doi: 10.3201/eid2108.141364, PMID: 26197993 PMC4517704

[ref45] Itoh Y. Rice J. D. Goller C. Pannuri A. Taylor J. Meisner J.. (2008). Roles of pgaabcd genes in synthesis, modification, and export of the *Escherichia coli* biofilm adhesin poly-beta-1,6-N-acetyl-D-glucosamine. J. Bacteriol. 190, 3670–3680. doi: 10.1128/JB.01920-07, PMID: 18359807 PMC2394981

[ref46] Kang S. Ravensdale J. Coorey R. Dykes G. A. Barlow R. (2019). A comparison of 16S rrna profiles through slaughter in Australian export beef abattoirs. Front. Microbiol. 10:2747. doi: 10.3389/fmicb.2019.02747, PMID: 31849891 PMC6895009

[ref47] Kang S. Ravensdale J. T. Coorey R. Dykes G. A. Barlow R. S. (2020). Bacterial community analysis using 16S rrna amplicon sequencing in the boning room of Australian beef export abattoirs. Int. J. Food Microbiol. 332:108779. doi: 10.1016/j.ijfoodmicro.2020.108779, PMID: 32673761

[ref48] Keenan D. F. Hayes J. E. Kenny T. A. Kerry J. P. (2016). Effect of hot boning and elevated brine temperature on the processing, storage and eating quality of cured beef hindquarter (M. Biceps femoris) and forequarter (*M. pectoralis* profundus) muscles. J. Food Qual. 39, 126–139. doi: 10.1111/jfq.12179

[ref49] Kempf F. La Ragione R. Chirullo B. Schouler C. Velge P. (2022). Super shedding in enteric pathogens: a review. Microorganisms 10:2101. doi: 10.3390/microorganisms10112101 36363692 PMC9692634

[ref50] Khaitsa M. L. Smith D. R. Stoner J. A. Parkhurst A. M. Hinkley S. Klopfenstein T. J.. (2003). Incidence, duration, and prevalence of *Escherichia coli* O157:H7 fecal shedding by feedlot cattle during the finishing period. J. Food Prot. 66, 1972–1977. doi: 10.4315/0362-028X-66.11.1972, PMID: 14627271

[ref51] Kim Y. Wang X. Ma Q. Zhang X. S. Wood T. K. (2009). Toxin-antitoxin systems in *Escherichia coli* influence biofilm formation through YjgK (TabA) and fimbriae. J. Bacteriol. 191, 1258–1267. doi: 10.1128/JB.01465-08, PMID: 19060153 PMC2632003

[ref52] King T. Kocharunchitt C. Gobius K. Bowman J. P. Ross T. (2016). Physiological response of *Escherichia coli* O157:H7 Sakai to dynamic changes in temperature and water activity as experienced during carcass chilling. Mol. Cell. Proteomics 15, 3331–3347. doi: 10.1074/mcp.M116.063065, PMID: 27615263 PMC5098033

[ref53] Kocharunchitt C. Mellefont L. Bowman J. P. Ross T. (2020). Application of chlorine dioxide and peroxyacetic acid during spray chilling as a potential antimicrobial intervention for beef carcasses. Food Microbiol. 87:103355. doi: 10.1016/j.fm.2019.103355, PMID: 31948612

[ref54] Langmead B. Salzberg S. L. (2012). Fast gapped-read alignment with bowtie 2. Nat. Methods 9, 357–359. doi: 10.1038/nmeth.1923, PMID: 22388286 PMC3322381

[ref55] Lavilla Lerma L. Benomar N. Casado Muñoz M. d. C. Gálvez A. Abriouel H. (2015). Correlation between antibiotic and biocide resistance in mesophilic and psychrotrophic Pseudomonas spp. isolated from slaughterhouse surfaces throughout meat chain production. Food Microbiol. 51, 33–44. doi: 10.1016/j.fm.2015.04.010, PMID: 26187825

[ref56] Lin Y. Yu C. Ma Z. Che L. Feng B. Fang Z.. (2022). Effects of yeast culture supplementation in wheat-rice-based diet on growth performance, meat quality, and gut microbiota of growing-finishing pigs. Animals 12:2177. doi: 10.3390/ani12172177 36077898 PMC9454582

[ref57] Liu N. T. Lefcourt A. M. Nou X. Shelton D. R. Zhang G. Lo Y. M. (2013). Native microflora in fresh-cut produce processing plants and their potentials for biofilm formation. J. Food Prot. 76, 827–832. doi: 10.4315/0362-028X.JFP-12-433, PMID: 23643124

[ref58] Liu Y. Youssef M. K. Yang X. (2016). Effects of dry chilling on the microflora on beef carcasses at a Canadian beef packing plant. J. Food Prot. 79, 538–543. doi: 10.4315/0362-028X.JFP-15-476, PMID: 27052856

[ref59] Lu J. Breitwieser F. P. Thielen P. Salzberg S. L. (2017). Bracken: estimating species abundance in metagenomics data. PeerJ Computer Science 3:e104. doi: 10.7717/peerj-cs.104 PMC12016282 40271438

[ref60] Mallick H. Rahnavard A. Mciver L. J. Ma S. Zhang Y. Nguyen L. H.. (2021). Multivariable association discovery in population-scale meta-omics studies. PLoS Comput. Biol. 17:e1009442. doi: 10.1371/journal.pcbi.1009442, PMID: 34784344 PMC8714082

[ref61] Mao S. Zhang M. Liu J. Zhu W. (2015). Characterising the bacterial microbiota across the gastrointestinal tracts of dairy cattle: membership and potential function. Sci. Rep. 5:16116. doi: 10.1038/srep16116, PMID: 26527325 PMC4630781

[ref62] Martin M. (2011). Cutadapt removes adapter sequences from high-throughput sequencing reads. EMBnet J. 2011, 3:10. doi: 10.14806/ej.17.1.200

[ref63] Maslen B. N. Gray L. A. Ghorashi S. A. White J. D. Campbell M. A. Pant S. D. (2022). Temporal changes in the faecal microbiota of beef cattle on feedlot placement. Animals 12:2500. doi: 10.3390/ani12192500 36230241 PMC9559285

[ref64] May T. Okabe S. (2011). Enterobactin is required for biofilm development in reduced-genome *Escherichia coli*. Environ. Microbiol. 13, 3149–3162. doi: 10.1111/j.1462-2920.2011.02607.x, PMID: 21980953

[ref65] Mcmurdie P. J. Holmes S. (2013). Phyloseq: an R package for reproducible interactive analysis and graphics of microbiome census data. PloS One 8:e61217. doi: 10.1371/journal.pone.0061217, PMID: 23630581 PMC3632530

[ref66] Meziti A. Rodriguez R. L. Hatt J. K. Pena-Gonzalez A. Levy K. Konstantinidis K. T. (2021). The reliability of metagenome-assembled genomes (mags) in representing natural populations: insights from comparing mags against isolate genomes derived from the same fecal sample. Appl. Environ. Microbiol. 87:20. doi: 10.1128/AEM.02593-20, PMID: 33452027 PMC8105024

[ref67] Møretrø T. Langsrud S. Heir E. (2013). Bacteria on meat abattoir process surfaces after sanitation: characterisation of survival properties of Listeria monocytogenes and the commensal bacterial flora. Adv. Microbiol. 3, 255–264. doi: 10.4236/aim.2013.33037

[ref68] Morimatsu K. Eguchi K. Hamanaka D. Tanaka F. Uchino T. (2012). Effects of temperature and nutrient conditions on biofilm formation of *Pseudomonas putida*. Food Sci. Technol. Res. 18, 879–883. doi: 10.3136/fstr.18.879

[ref69] Nan Y. Rodas-Gonzalez A. Stanford K. Nadon C. Yang X. Mcallister T.. (2022). Formation and transfer of multi-species biofilms Containing *E. coli* O103:H2 on food contact surfaces to beef. Front. Microbiol. 13:863778. doi: 10.3389/fmicb.2022.863778, PMID: 35711784 PMC9196126

[ref70] Narvaez-Bravo C. Miller M. F. Jackson T. Jackson S. Rodas-Gonzalez A. Pond K.. (2013). Salmonella and *Escherichia coli* O157:H7 prevalence in cattle and on carcasses in a vertically integrated feedlot and harvest plant in Mexico. J. Food Prot. 76, 786–795. doi: 10.4315/0362-028X.JFP-12-079, PMID: 23643120

[ref71] Nastasijevic I. Schmidt J. W. Boskovic M. Glisic M. Kalchayanand N. Shackelford S. D.. (2020). Seasonal prevalence of Shiga toxin-producing *Escherichia coli* on pork carcasses for three steps of the harvest process at two commercial processing plants in the United States. Appl. Environ. Microbiol. 87:20. doi: 10.1128/AEM.01711-20, PMID: 33067201 PMC7755256

[ref72] Niba E. T. Naka Y. Nagase M. Mori H. Kitakawa M. (2007). A genome-wide approach to identify the genes involved in biofilm formation in *E. coli*. DNA Res. 14, 237–246. doi: 10.1093/dnares/dsm024, PMID: 18180259 PMC2779908

[ref73] O’Toole G. Kaplan H. B. Kolter R. (2000). Biofilm formation as microbial development. Annu. Rev. Microbiol. 54, 49–79. doi: 10.1146/annurev.micro.54.1.49 11018124

[ref74] O’Toole G. A. Kolter R. (1998). Flagellar and twitching motility are necessary for *Pseudomonas aeruginosa* biofilm development. Mol. Microbiol. 30, 295–304. doi: 10.1046/j.1365-2958.1998.01062.x, PMID: 9791175

[ref75] Overbeek R. Begley T. Butler R. M. Choudhuri J. V. Chuang H. Y. Cohoon M.. (2005). The subsystems approach to genome annotation and its use in the project to annotate 1000 genomes. Nucleic Acids Res. 33, 5691–5702. doi: 10.1093/nar/gki866, PMID: 16214803 PMC1251668

[ref76] Overbeek R. Olson R. Pusch G. D. Olsen G. J. Davis J. J. Disz T.. (2014). The seed and the rapid annotation of microbial genomes using subsystems technology (Rast). Nucleic Acids Res. 42, D206–D214. doi: 10.1093/nar/gkt1226, PMID: 24293654 PMC3965101

[ref77] Paranjpye R. N. Strom M. S. (2005). A *Vibrio vulnificus* type iv pilin contributes to biofilm formation, adherence to epithelial cells, and virulence. Infect. Immun. 73, 1411–1422. doi: 10.1128/IAI.73.3.1411-1422.2005, PMID: 15731039 PMC1064924

[ref78] Quan J. Wu Z. Ye Y. Peng L. Wu J. Ruan D.. (2020). Metagenomic sharacterization of tntestinal regions in pigs with contrasting feed efficiency. Front. Microbiol. 11:32. doi: 10.3389/fmicb.2020.00032, PMID: 32038603 PMC6989599

[ref79] Quast C. Pruesse E. Yilmaz P. Gerken J. Schweer T. Yarza P.. (2013). The Silva ribosomal Rna gene database project: improved data processing and web-based tools. Nucleic Acids Res. 41, D590–D596. doi: 10.1093/nar/gks1219, PMID: 23193283 PMC3531112

[ref80] Römling U. Galperin M. Y. (2015). Bacterial cellulose biosynthesis: diversity of operons, subunits, products, and functions. Trends Microbiol. 23, 545–557. doi: 10.1016/j.tim.2015.05.005 26077867 PMC4676712

[ref81] Savell J. W. Mueller S. L. Baird B. E. (2005). The chilling of carcasses. Meat Sci. 70, 449–459. doi: 10.1016/j.meatsci.2004.06.027 22063744

[ref82] Scott B. R. Yang X. Geornaras I. Delmore R. J. Woerner D. R. Adler J. M.. (2015). Antimicrobial efficacy of a lactic acid and citric acid blend against Shiga toxin-producing *Escherichia coli*, Salmonella, and nonpathogenic *Escherichia coli* biotype I on inoculated prerigor beef carcass surface tissue. J. Food Prot. 78, 2136–2142. doi: 10.4315/0362-028X.JFP-15-194 26613907

[ref83] Shaw M. K. Marr A. G. Ingraham J. L. (1971). Determination of the minimal temperature for growth of *Escherichia coli*. J. Bacteriol. 105, 683–684. doi: 10.1128/jb.105.2.683-684.1971, PMID: 4925195 PMC248456

[ref84] Shedleur-Bourguignon F. Duchemin T. Thériault W. Longpré J. Thibodeau A. Hocine M. N.. (2023). Distinct microbiotas are associated with different production lines in the cutting room of a swine slaughterhouse. Microorganisms 11:133. doi: 10.3390/microorganisms11010133, PMID: 36677425 PMC9862343

[ref85] Smith M. G. (1985). The generation time, lag time, and minimum temperature of growth of coliform organisms on meat, and the implications for codes of practice in abattoirs. J. Hyg. 94, 289–300. doi: 10.1017/S0022172400061519, PMID: 3891847 PMC2129483

[ref86] Stanford K. Tran F. Zhang P. Yang X. (2021). Biofilm-forming capacity of *Escherichia coli* isolated from cattle and beef packing plants: relation to virulence attributes, stage of processing, antimicrobial interventions, and heat tolerance. Appl. Environ. Microbiol. 87:e0112621. doi: 10.1128/AEM.01126-21, PMID: 34550756 PMC8579979

[ref87] Stephens T. P. Mcallister T. A. Stanford K. (2009). Perineal swabs reveal effect of super shedders on the transmission of *Escherichia coli* O157:H7 in commercial feedlots. J. Anim. Sci. 87, 4151–4160. doi: 10.2527/jas.2009-1967, PMID: 19684276

[ref88] Tarr P. I. Gordon C. A. Chandler W. L. (2005). Shiga-toxin-producing Escherichia coli and haemolytic uraemic syndrome. Lancet 365, 1073–1086. doi: 10.1016/S0140-6736(05)71144-2, PMID: 15781103

[ref89] Thorpe C. M. (2004). Shiga toxin-producing *Escherichia coli* infection. Clin. Infect. Dis. 38, 1298–1303. doi: 10.1086/383473 15127344

[ref90] Uhlich G. A. Chen C. Y. Cottrell B. J. Hofmann C. S. Dudley E. G. Strobaugh T. P.. (2013). Phage insertion in mlrA and variations in rpoS limit curli expression and biofilm formation in *Escherichia coli* serotype O157: H7. Microbiology 159, 1586–1596. doi: 10.1099/mic.0.066118-0, PMID: 23744902

[ref91] Uhlich G. A. Chen C.-Y. Cottrell B. J. Nguyen L.-H. (2014). Growth media and temperature effects on biofilm formation by serotype O157:H7 and non-O157 Shiga toxin-producing *Escherichia coli*. FEMS Microbiol. Lett. 354, 133–141. doi: 10.1111/1574-6968.12439 24702283

[ref92] Van Houdt R. Michiels C. W. (2005). Role of bacterial cell surface structures in *Escherichia coli* biofilm formation. Res. Microbiol. 156, 626–633. doi: 10.1016/j.resmic.2005.02.005 15950122

[ref93] Visvalingam J. Wang H. Youssef M. K. Devos J. Gill C. O. Yang X. (2016). Spatial and temporal distribution of *Escherichia coli* on beef trimmings obtained from a beef packing plant. J. Food Prot. 79, 1325–1331. doi: 10.4315/0362-028X.JFP-15-598, PMID: 27497119

[ref94] Visvalingam J. Zhang P. Ells T. C. Yang X. (2019). Dynamics of biofilm formation by Salmonella typhimurium and beef processing plant bacteria in mono- and dual-species cultures. Microb. Ecol. 78, 375–387. doi: 10.1007/s00248-018-1304-z, PMID: 30547194

[ref95] Wagner E. M. Fischel K. Rammer N. Beer C. Palmetzhofer A. L. Conrady B.. (2021). Bacteria of eleven different species isolated from biofilms in a meat processing environment have diverse biofilm forming abilities. Int. J. Food Microbiol. 349:109232. doi: 10.1016/j.ijfoodmicro.2021.109232 34022615

[ref96] Wang R. (2019). Biofilms and meat safety: a mini-review. J. Food Prot. 82, 120–127. doi: 10.4315/0362-028X.JFP-18-311 30702946

[ref97] Wang R. Bono J. L. Kalchayanand N. Shackelford S. Harhay D. M. (2012). Biofilm formation by Shiga toxin-producing *Escherichia coli* O157:H7 and non-O157 strains and their tolerance to sanitizers commonly used in the food processing environment. J. Food Prot. 75, 1418–1428. doi: 10.4315/0362-028X.JFP-11-427 22856565

[ref98] Wang H. He A. Yang X. (2018). Dynamics of microflora on conveyor belts in a beef fabrication facility during sanitation. Food Control 85, 42–47. doi: 10.1016/j.foodcont.2017.09.017

[ref99] Wang R. Luedtke B. E. Bosilevac J. M. Schmidt J. W. Kalchayanand N. Arthur T. M. (2016). *Escherichia coli* O157:H7 strains isolated from high-event period beef contamination have strong biofilm-forming ability and low sanitizer susceptibility, which are associated with high pO157 plasmid copy number. J. Food Prot. 79, 1875–1883. doi: 10.4315/0362-028X.JFP-16-113, PMID: 28221917

[ref100] Watnick P. Kolter R. (2000). Biofilm, City of microbes. J. Bacteriol. 182, 2675–2679. doi: 10.1128/JB.182.10.2675-2679.2000, PMID: 10781532 PMC101960

[ref101] White D. Drummond J. Fuqua C. (2011). The physiology and biochemistry of prokaryotes. Oxford, United Kingdom: Oxford University Press.

[ref102] WHO (2018). Shiga toxin-producing *Escherichia coli* (Stec) and food. Microbiological risk assessment series; 31. Rome: World Health Organization.

[ref103] Wickham H. (2016). Ggplot2: Elegant graphics for data analysis, Springer-Verlag New York.

[ref104] Wickramasinghe N. N. Hlaing M. M. Ravensdale J. T. Coorey R. Chandry P. S. Dykes G. A. (2020). Characterization of the biofilm matrix composition of psychrotrophic, meat spoilage pseudomonads. Sci. Rep. 10:16457. doi: 10.1038/s41598-020-73612-0, PMID: 33020559 PMC7536239

[ref105] Wood D. E. Lu J. Langmead B. (2019). Improved metagenomic analysis with kraken 2. Genome Biol. 20:257. doi: 10.1186/s13059-019-1891-0 31779668 PMC6883579

[ref106] Xu Z. S. Zhu T. Wang Z. Yang X. Gänzle M. G. (2023). Socializing at the air-liquid tnterface: a functional genomic analysis on biofilm-related genes during pellicle formation by Escherichia coli and its interaction with Aeromonas australiensis. Appl. Environ. Microbiol. 89:e0045623. doi: 10.1128/aem.00456-23, PMID: 37310210 PMC10370300

[ref107] Yang X. (2017). “Microbial ecology of beef carcasses and beef products” in Quantitative Microbiology Food Processing. John Wiley & Sons, Ltd, the Atrium, Southern Gate, Chichester, West Sussex, PO19 8SQ, UK.

[ref108] Yang X. Badoni M. Tran F. Gill C. O. (2015b). Microbiological effects of a routine treatment for decontaminating hide-on carcasses at a large beef packing plant. J. Food Prot. 78, 256–263. doi: 10.4315/0362-028X.JFP-14-226 25710139

[ref109] Yang X. Badoni M. Youssef M. K. Gill C. O. (2012). Enhanced control of microbiological contamination of product at a large beef packing plant. J. Food Prot. 75, 144–149. doi: 10.4315/0362-028X.JFP-11-291 22221368

[ref110] Yang X. He A. Badoni M. Tran F. Wang H. (2017a). Mapping sources of contamination of *Escherichia coli* on beef in the fabrication facility of a commercial beef packing plant. Food Control 75, 153–159. doi: 10.1016/j.foodcont.2016.12.004

[ref111] Yang X. Noyes N. R. Doster E. Martin J. N. Linke L. M. Magnuson R. J.. (2016). Use of metagenomic shotgun sequencing technology to detect foodborne pathogens within the microbiome of the beef production chain. Appl. Environ. Microbiol. 82, 2433–2443. doi: 10.1128/AEM.00078-16 26873315 PMC4959480

[ref112] Yang X. Tran F. Wolters T. (2017b). Microbial ecology of decontaminated and not decontaminated beef carcasses. J. Food Res. 6, 85–91. doi: 10.5539/jfr.v6n5p85

[ref113] Yang X. Tran F. Youssef M. K. Gill C. O. (2015a). Determination of sources of *Escherichia coli* on beef by multiple-locus variable-number tandem repeat analysis. J. Food Prot. 78, 1296–1302. doi: 10.4315/0362-028X.JFP-15-014, PMID: 26197280

[ref114] Yang X. Tran F. Zhang P. (2023a). Comparative genomic analyses of *Escherichia coli* from a meat processing environment in relation to their biofilm formation and persistence. Microbiol Spectr 11:e0018323. doi: 10.1128/spectrum.00183-23, PMID: 37184412 PMC10269509

[ref115] Yang X. Wang H. He A. Tran F. (2017c). Microbial efficacy and impact on the population of *Escherichia coli* of a routine sanitation process for the fabrication facility of a beef packing plant. Food Control 71, 353–357. doi: 10.1016/j.foodcont.2016.07.016

[ref116] Yang X. Wang H. He A. Tran F. (2018). Biofilm formation and susceptibility to biocides of recurring and transient *Escherichia coli* isolated from meat fabrication equipment. Food Control 90, 205–211. doi: 10.1016/j.foodcont.2018.02.050

[ref117] Yang X. Wang H. Hrycauk S. Holman D. B. Ells T. C. (2023b). Microbial dynamics in mixed-culture biofilms of Salmonella typhimurium and *Escherichia coli* O157: H7 and bacteria surviving sanitation of conveyor belts of meat processing plants. Microorganisms 11:421. doi: 10.3390/microorganisms11020421, PMID: 36838386 PMC9960345

[ref118] Yang X. Wang H. Hrycauk S. Klassen M. D. (2021). Effects of peroxyacetic acid spray and storage temperature on the microbiota and sensory properties of vacuum-packed subprimal cuts of meat. Appl. Environ. Microbiol. 87:20. doi: 10.1128/AEM.03143-20, PMID: 33771784 PMC8208153

[ref119] Yin W. Wang Y. Liu L. He J. (2019). Biofilms: the microbial “protective clothing” in extreme environments. Int. J. Mol. Sci. 20:423. doi: 10.3390/ijms20143423, PMID: 31336824 PMC6679078

[ref120] Youssef M. K. Badoni M. Yang X. Gill C. O. (2013). Sources of *Escherichia coli* deposited on beef during breaking of carcasses carrying few *E. coli* at two packing plants. Food Control 31, 166–171. doi: 10.1016/j.foodcont.2012.09.045

[ref121] Youssef M. K. Gill C. O. Yang X. (2014). Storage life at 2oC or −1.5oC of vacuum-packaged boneless and bone-in cuts from decontaminated beef carcasses. J. Sci. Food Agric. 94, 3118–3124. doi: 10.1002/jsfa.6659 24647970

[ref122] Zaheer R. Lakin S. M. Polo R. O. Cook S. R. Larney F. J. Morley P. S.. (2019). Comparative diversity of microbiomes and Resistomes in beef feedlots, downstream environments and urban sewage influent. BMC Microbiol. 19:197. doi: 10.1186/s12866-019-1548-x, PMID: 31455230 PMC6712873

[ref123] Zdolec N. Kotsiri A. Houf K. Alvarez-Ordonez A. Blagojevic B. Karabasil N.. (2022). Systematic review and meta-analysis of the efficacy of interventions applied during primary processing to reduce microbial contamination on pig carcasses. Foods 11:110. doi: 10.3390/foods11142110 35885353 PMC9315615

[ref124] Zhang P. Badoni M. Gänzle M. Yang X. (2018). Growth of Carnobacterium spp. isolated from chilled vacuum-packaged meat under relevant acidic conditions. Int. J. Food Microbiol. 286, 120–127. doi: 10.1016/j.ijfoodmicro.2018.07.032 30081251

[ref125] Zhang J. Poh C. L. (2018). Regulating exopolysaccharide gene wcaF allows control of *Escherichia coli* biofilm formation. Sci. Rep. 8:13127. doi: 10.1038/s41598-018-31161-7 30177768 PMC6120894

[ref126] Zhang Z. Yang L. He Y. Luo X. Zhao S. Jia X. (2021). Composition of fecal microbiota in grazing and feedlot angus beef cattle. Animals 11:3167. doi: 10.3390/ani11113167 34827898 PMC8614352

[ref127] Zhilyaev S. Cadavez V. Gonzales-Barron U. Phetxumphou K. Gallagher D. (2017). Meta-analysis on the effect of interventions used in cattle processing plants to reduce *Escherichia coli* contamination. Food Res. Int. 93, 16–25. doi: 10.1016/j.foodres.2017.01.005, PMID: 28290276

[ref128] Zwirzitz B. Wetzels S. U. Dixon E. D. Fleischmann S. Selberherr E. Thalguter S.. (2021). Co-occurrence of Listeria spp. and spoilage associated microbiota during meat processing due to cross-contamination events. Front. Microbiol. 12:632935. doi: 10.3389/fmicb.2021.632935 33613505 PMC7892895

[ref129] Zwirzitz B. Wetzels S. U. Dixon E. D. Stessl B. Zaiser A. Rabanser I.. (2020). The sources and transmission routes of microbial populations throughout a meat processing facility. NPJ Biofilms Microbiomes 6:26. doi: 10.1038/s41522-020-0136-z, PMID: 32651393 PMC7351959

