# Supplementary material for: Driving forces shaping the microbial ecology in meat packing plants
Source: Front Microbiol. 2024 Jan 23;14:1333696. doi: 10.3389/fmicb.2023.1333696 (PMC10844536; doi:10.3389/fmicb.2023.1333696)

**Figure S1.** The correlations found in three or two functional rooms (after-cleaning). Both x and y axis show the log transformation of relative abundance of a bacterial genus, with -2, -1, 0, 1, and 2 referring to <0.1, 0.1, 1, 10, 100% respectively.


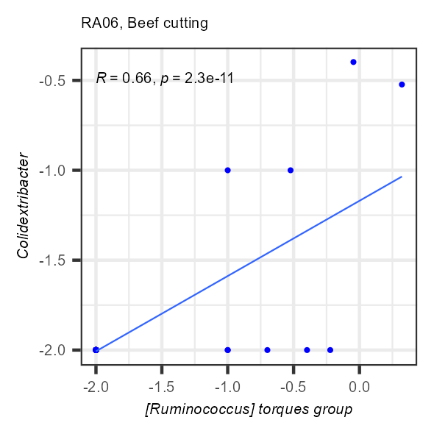

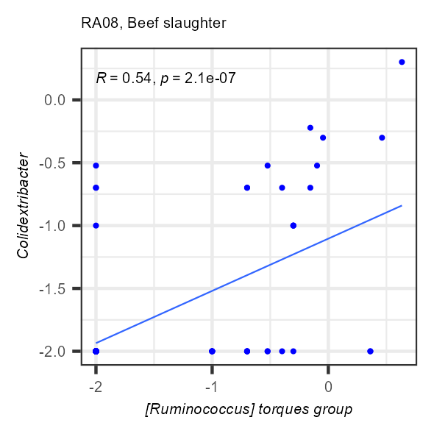

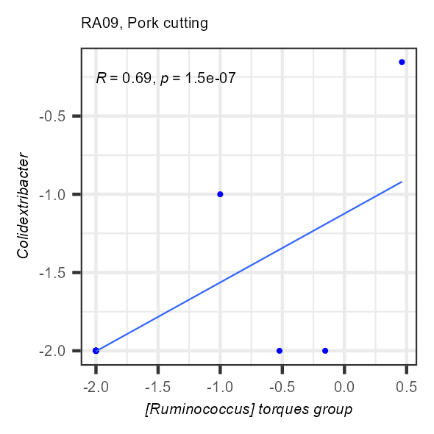

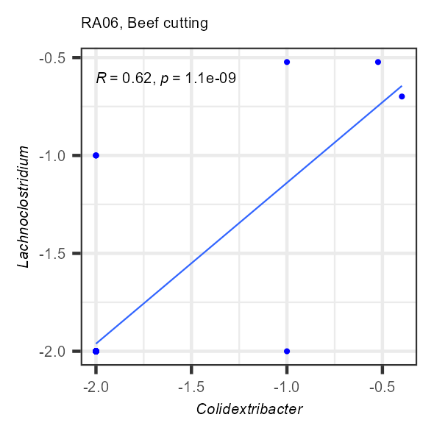

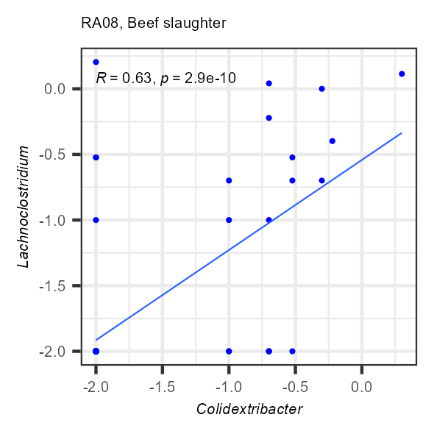

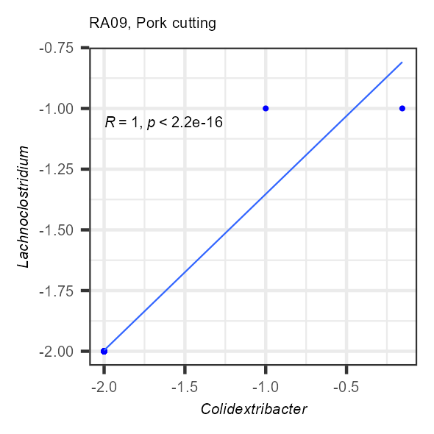


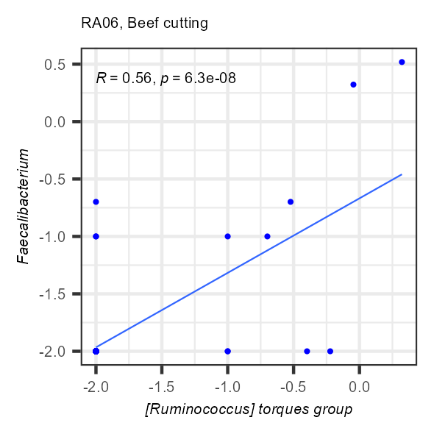

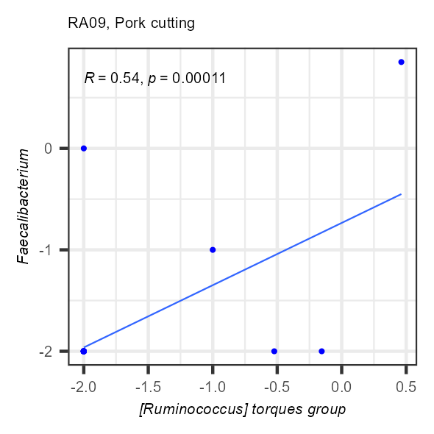


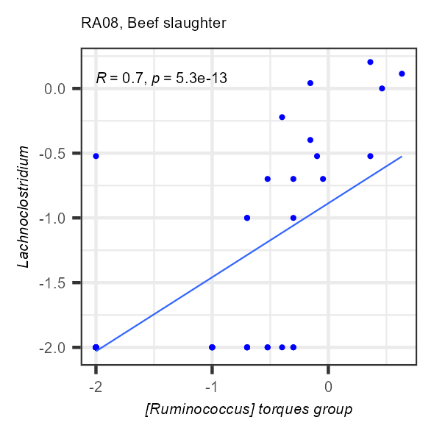

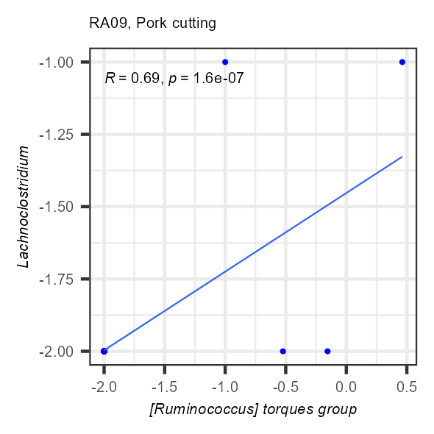


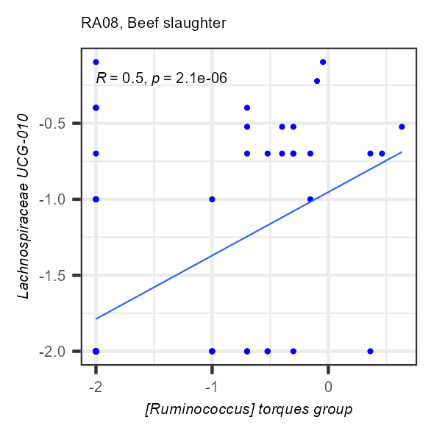

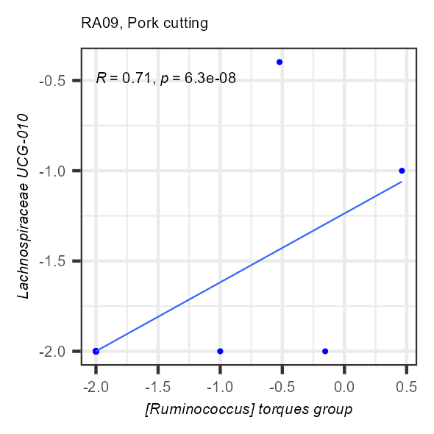


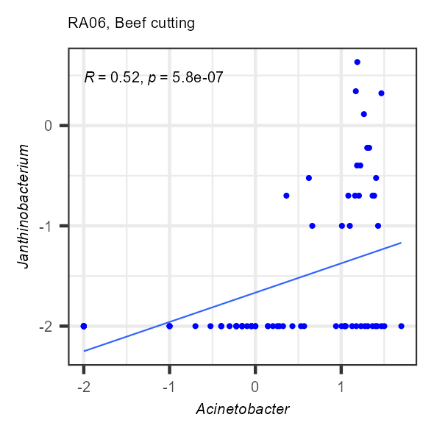

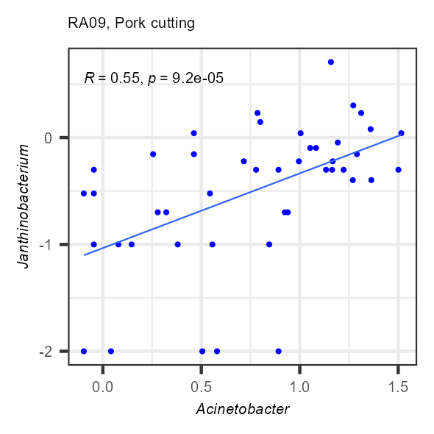


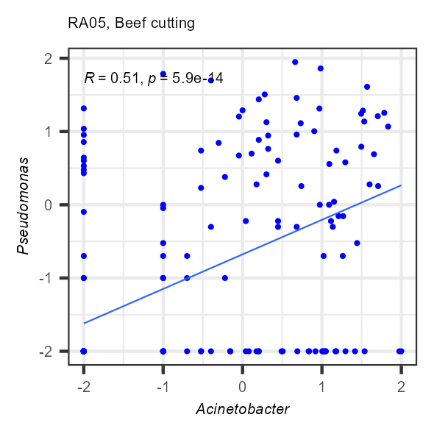

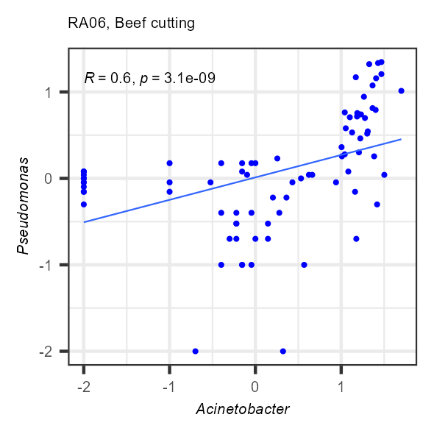


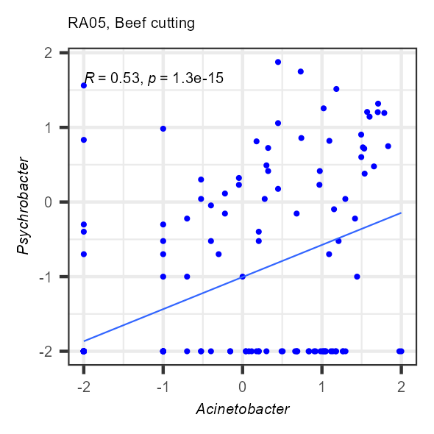

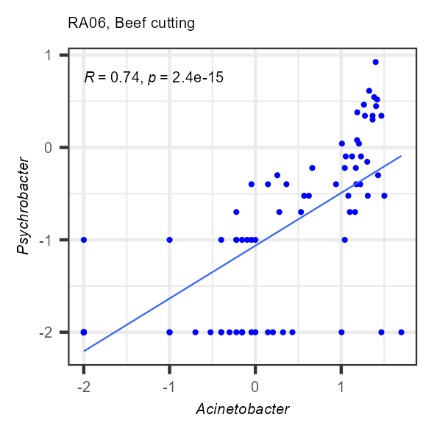


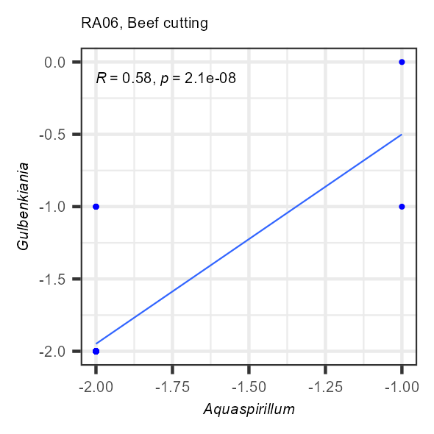

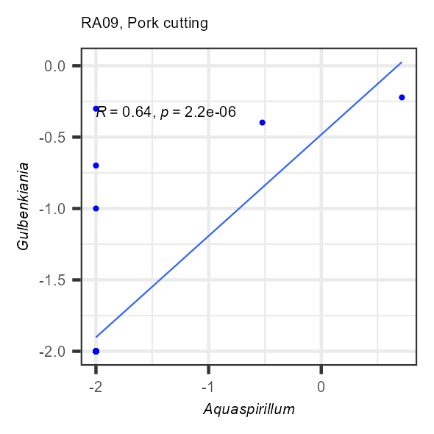


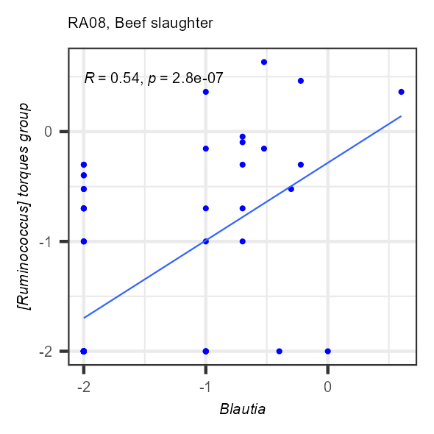

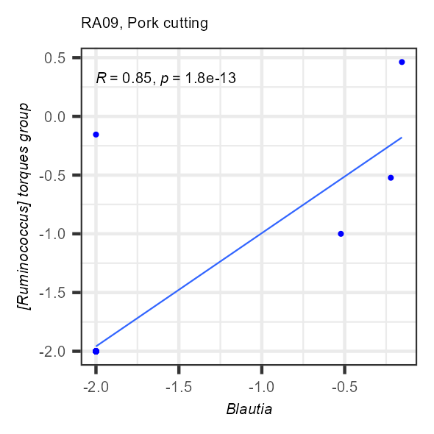


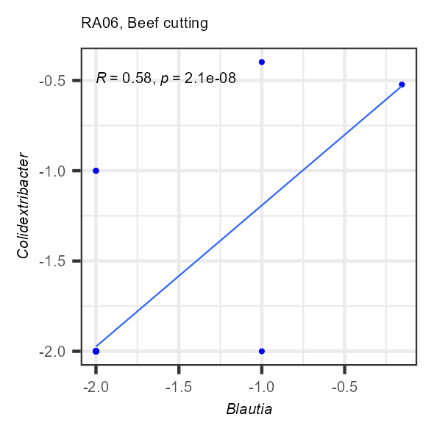

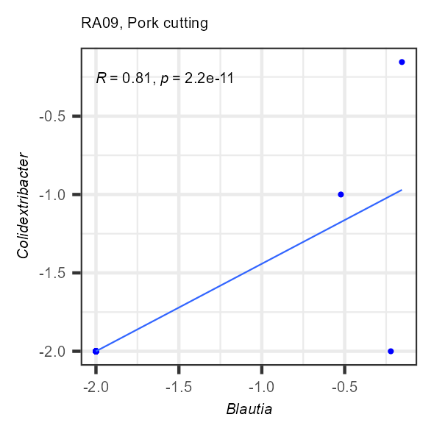


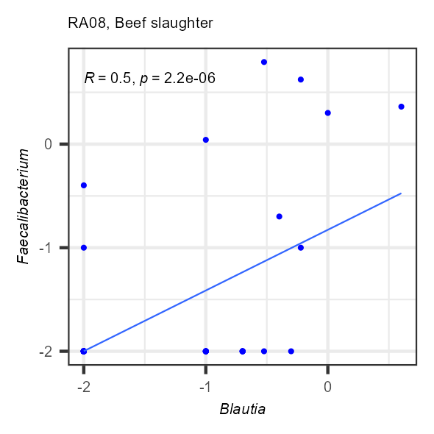

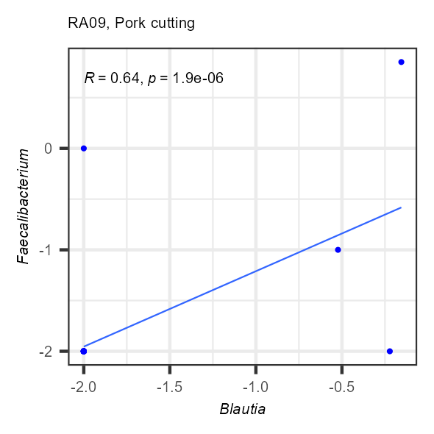


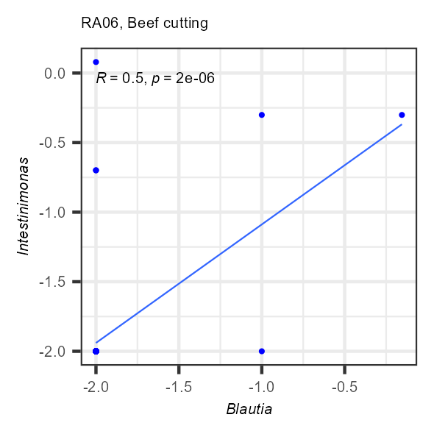

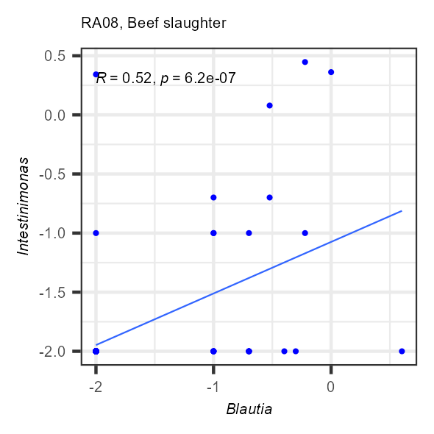


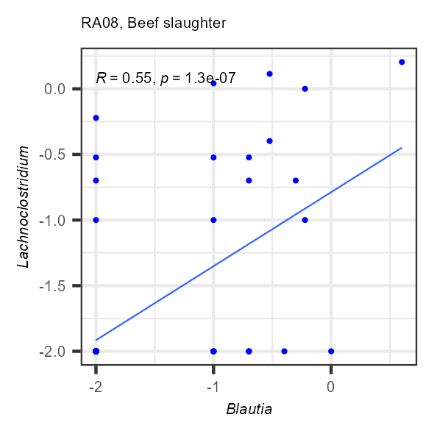

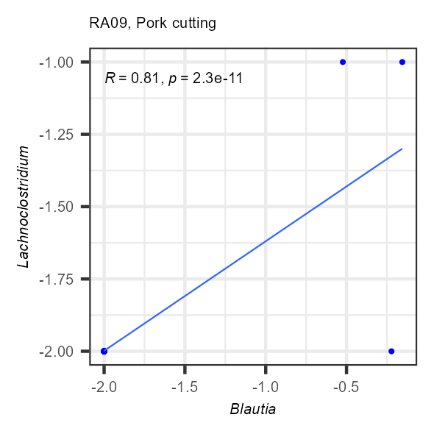


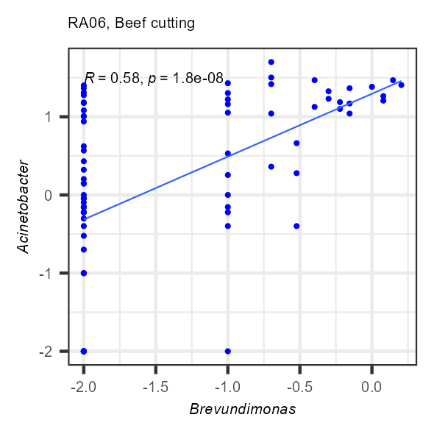

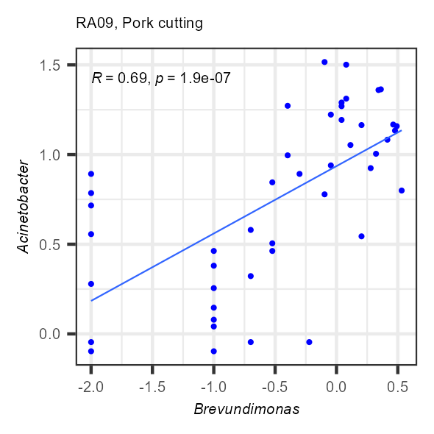


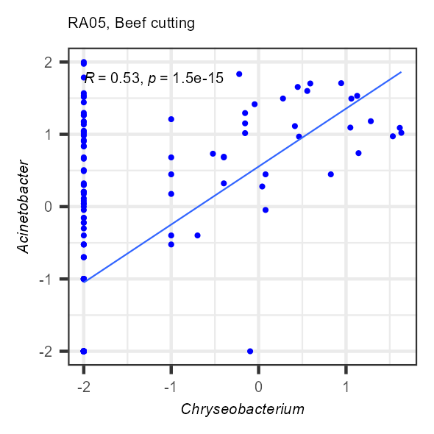

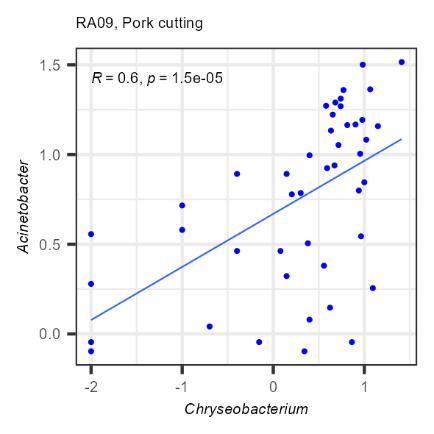


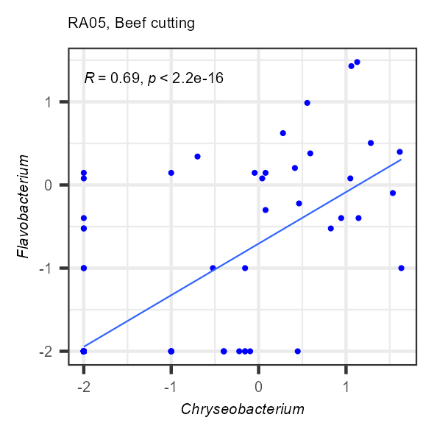

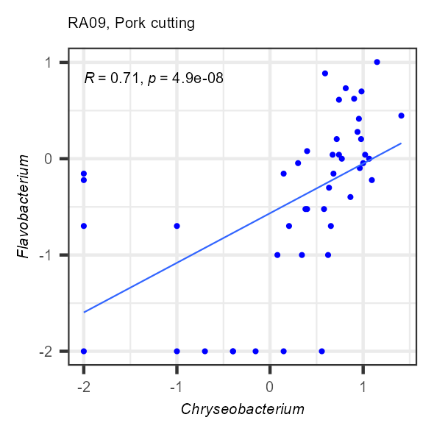


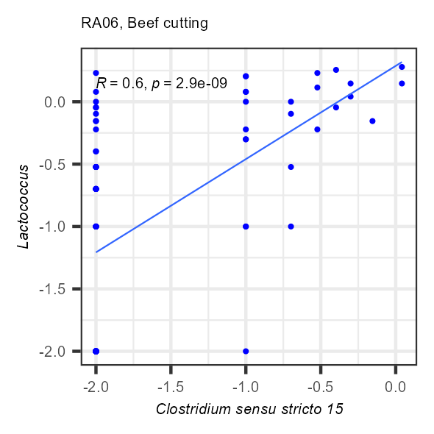

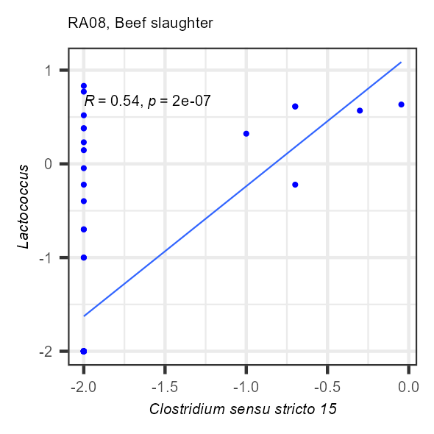


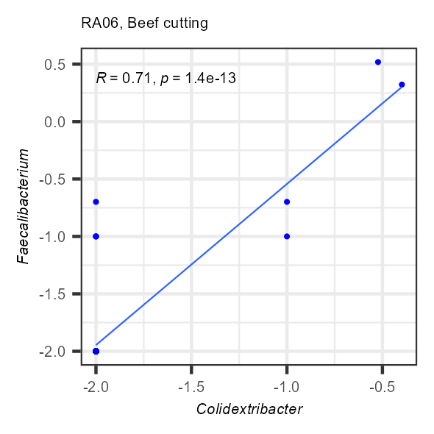

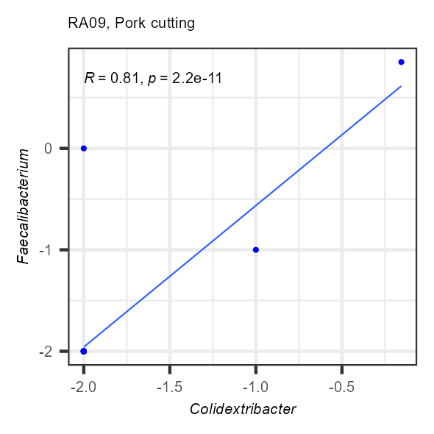


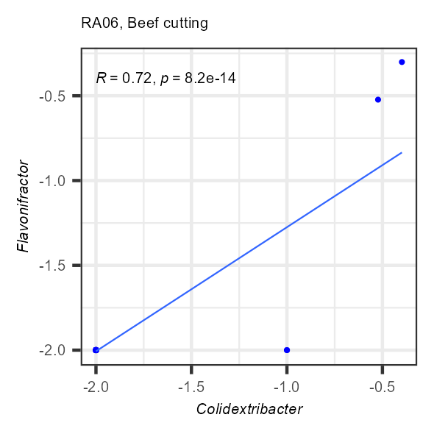

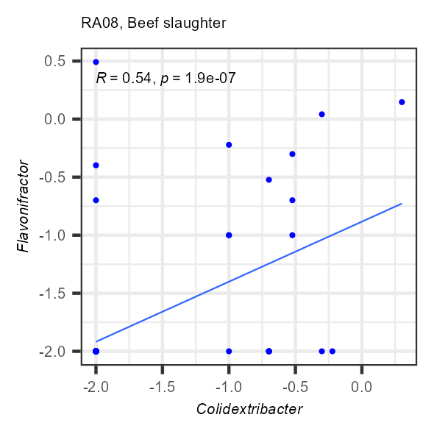


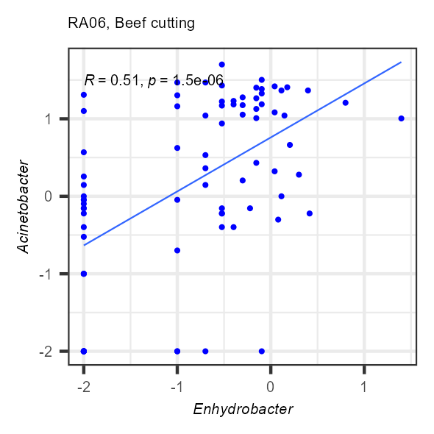

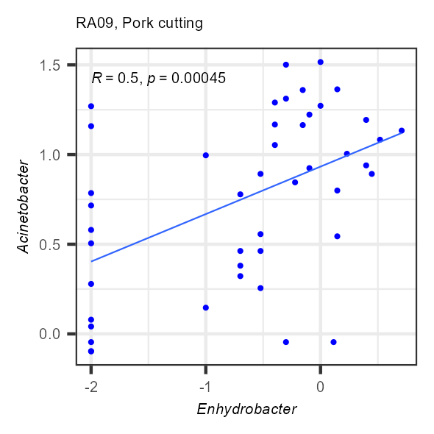


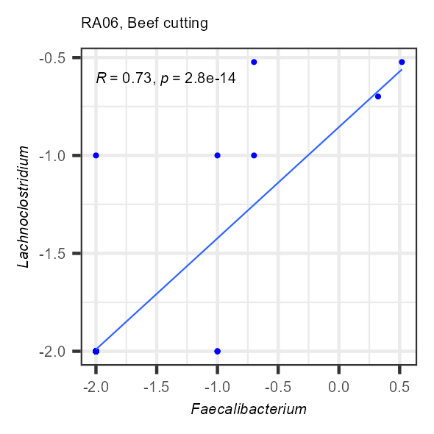

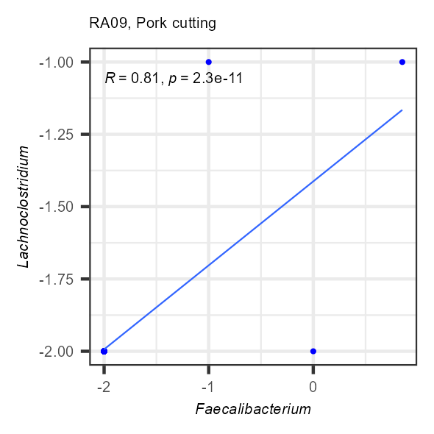


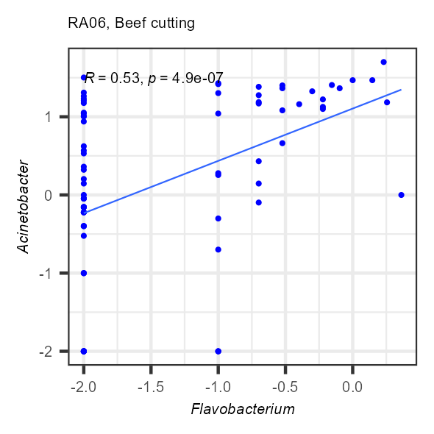

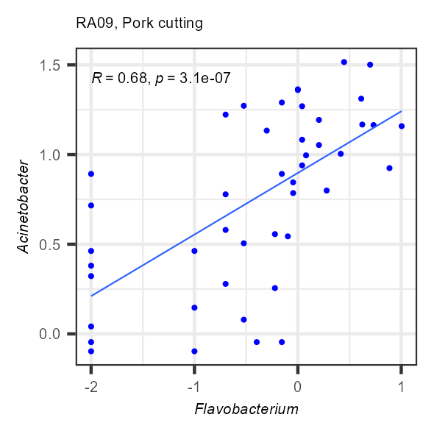


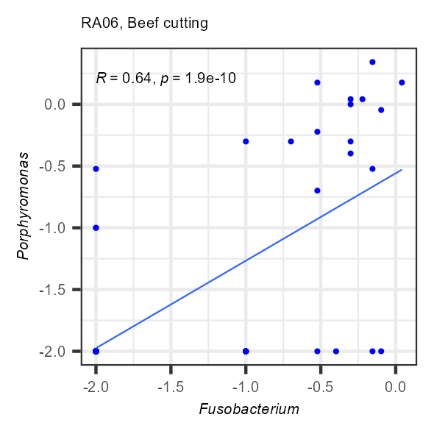

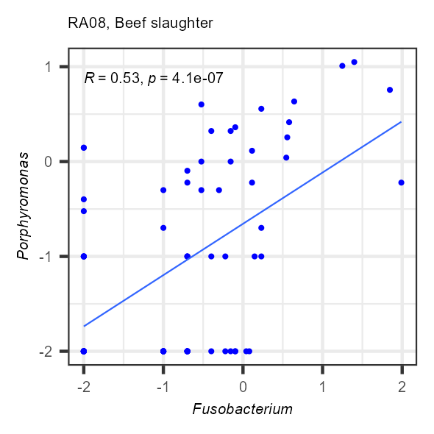


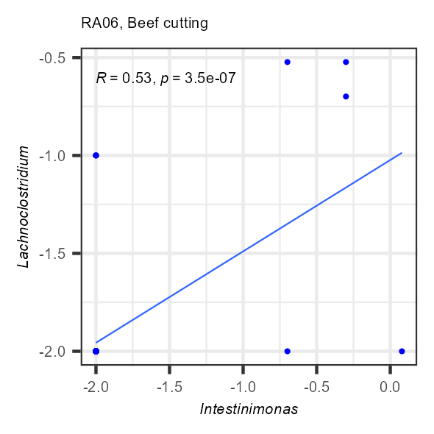

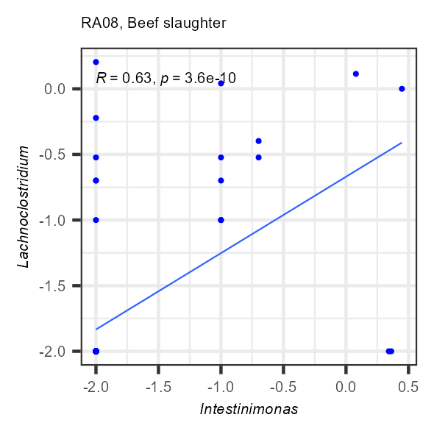


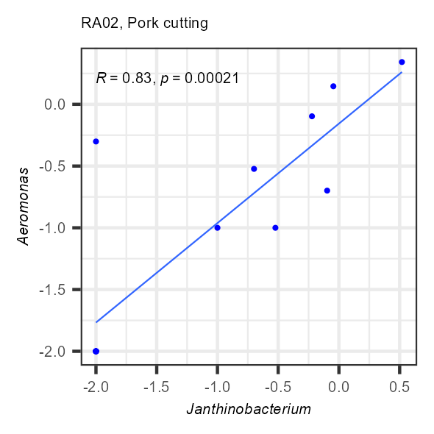

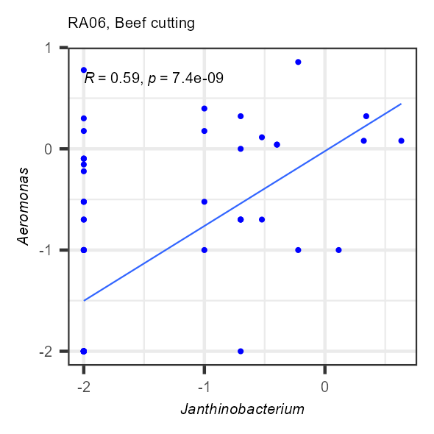


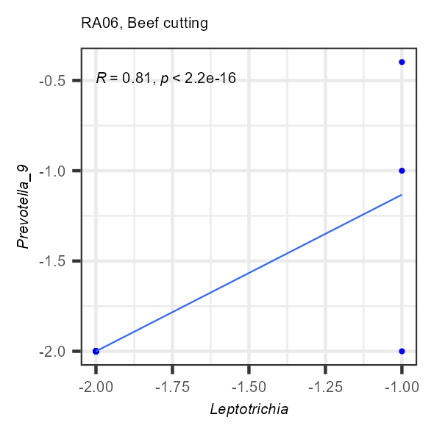

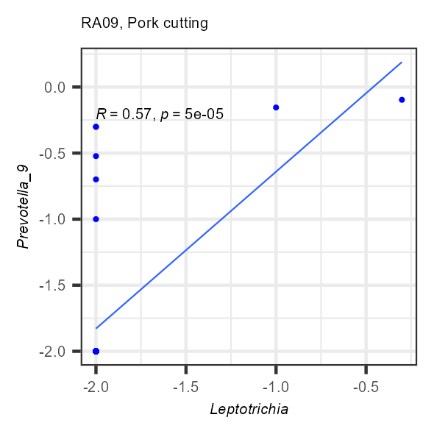


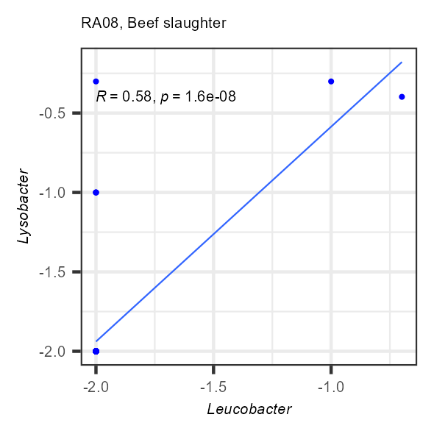

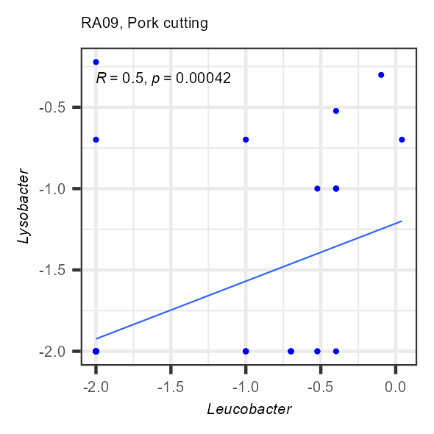


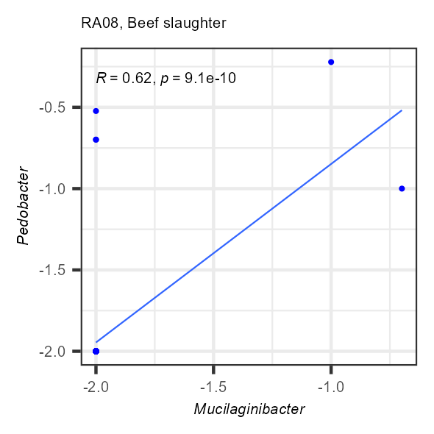

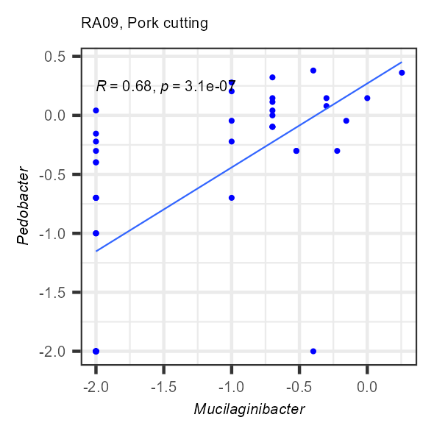


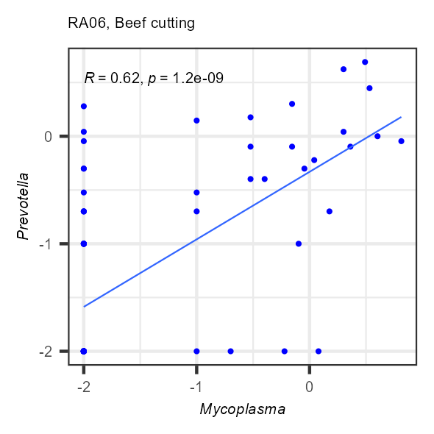

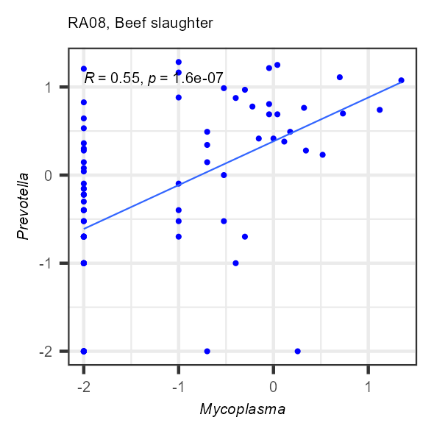


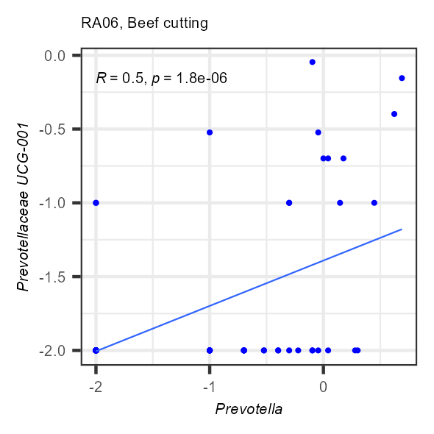

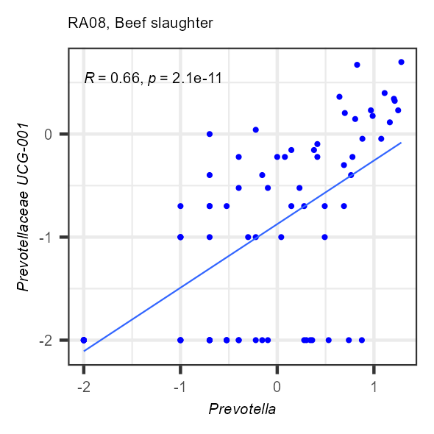


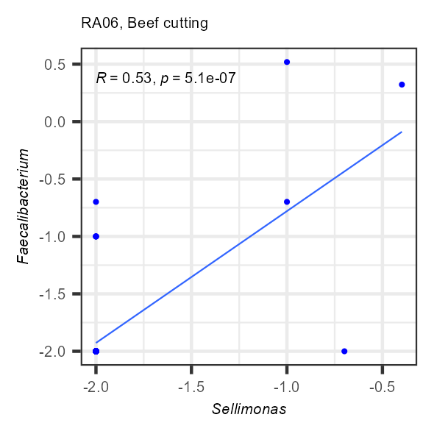

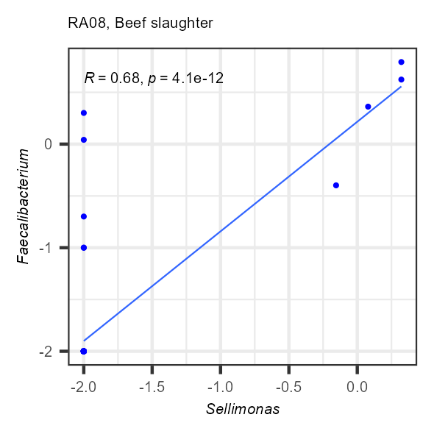


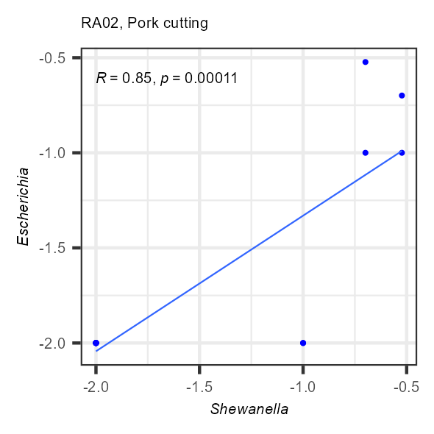

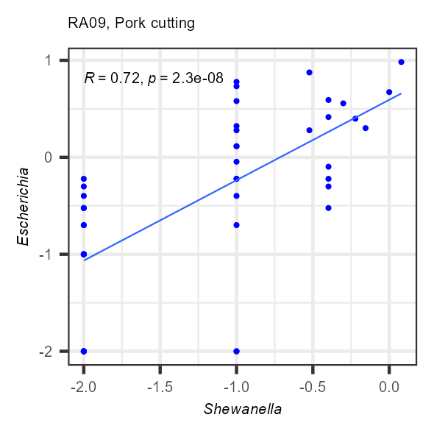


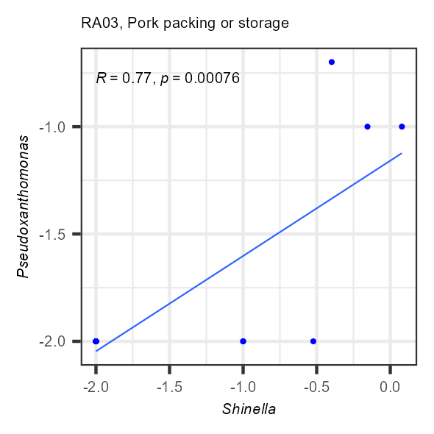

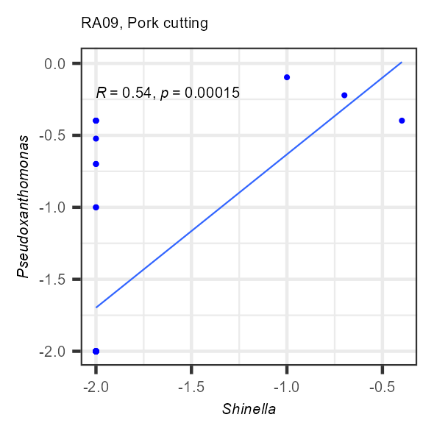


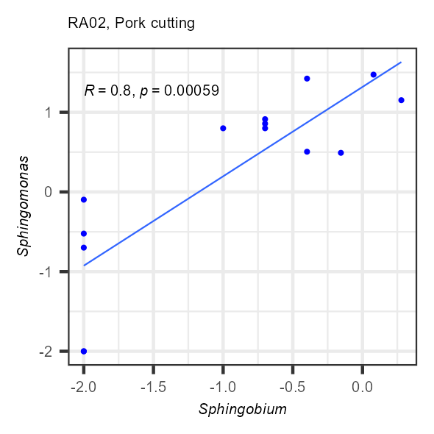

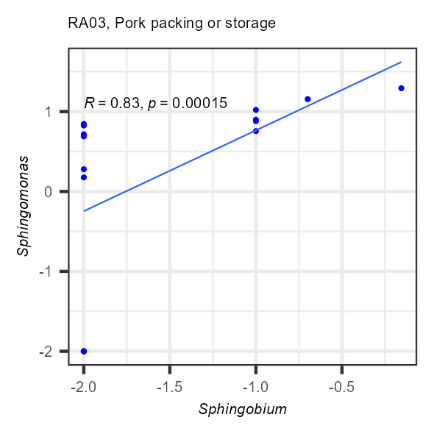


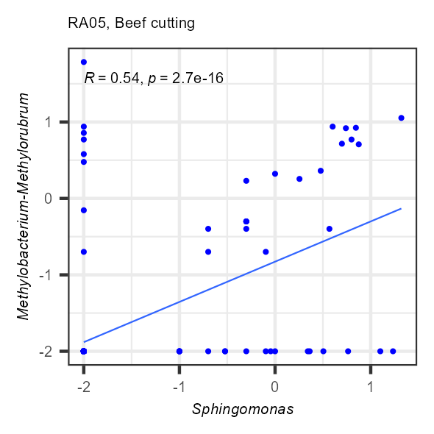

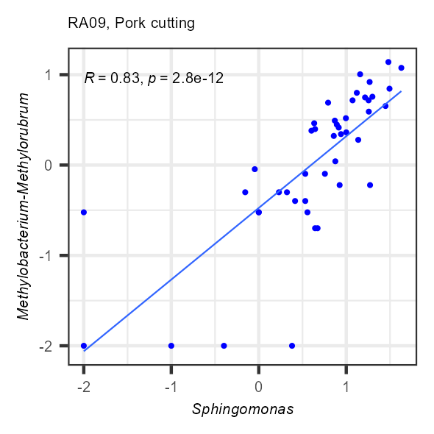


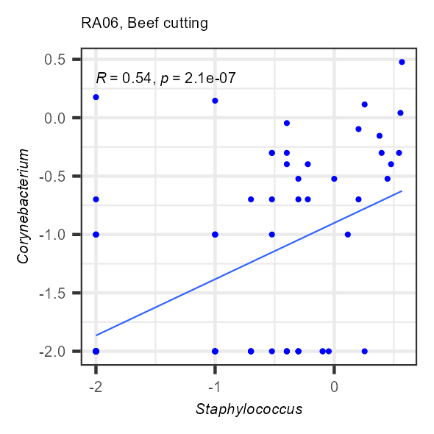

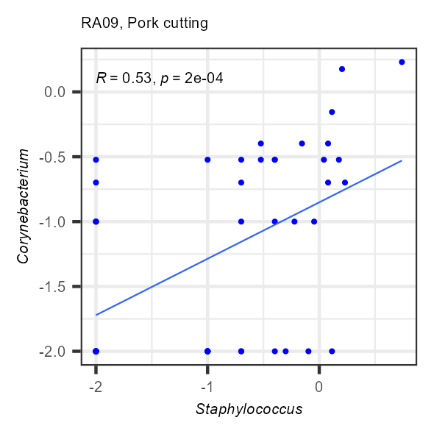


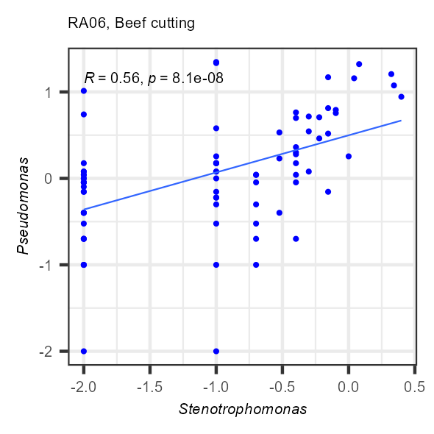

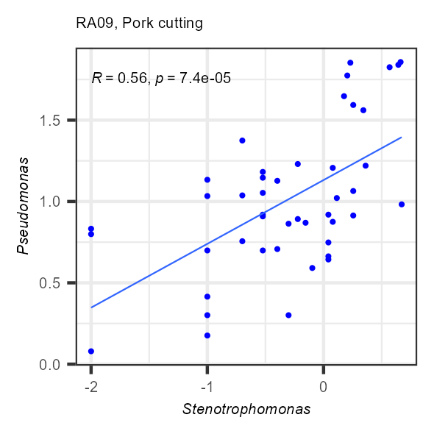


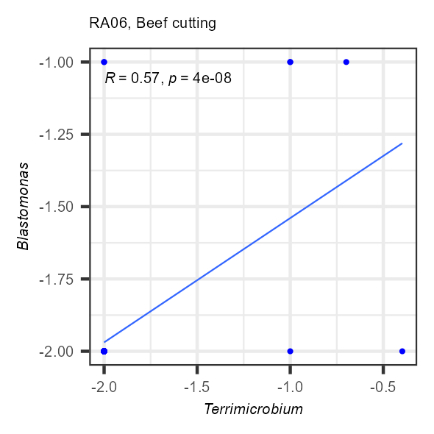

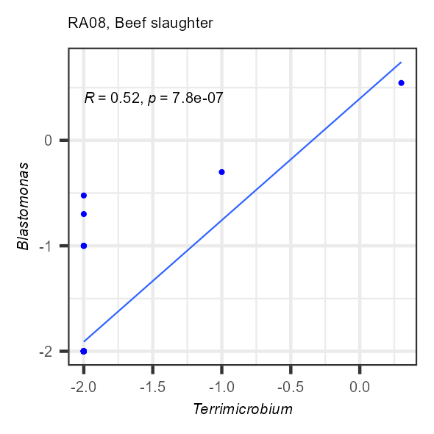


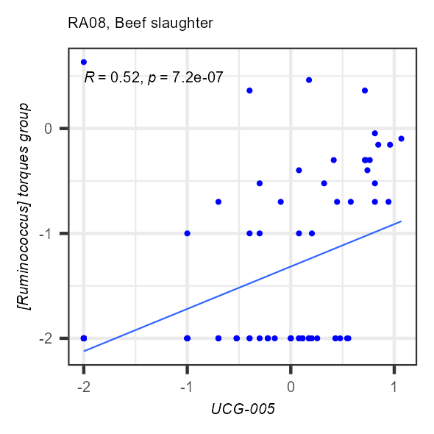

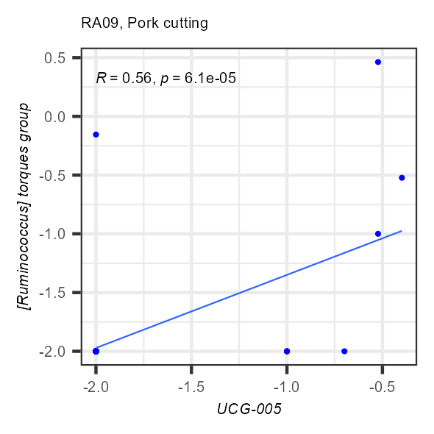


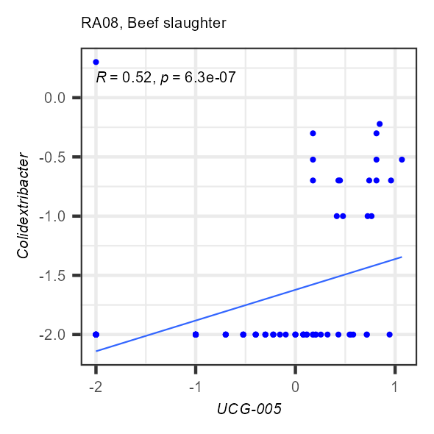

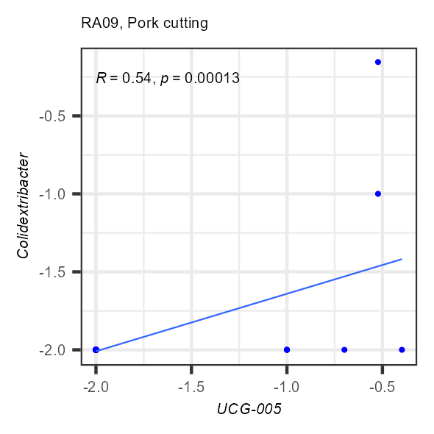


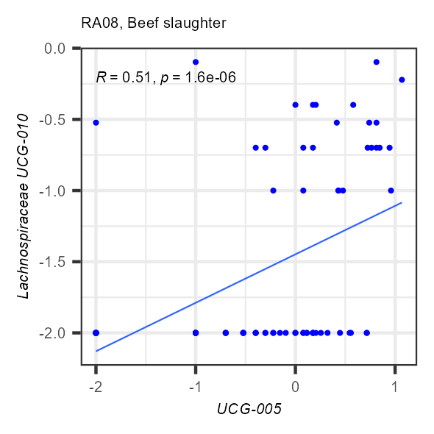

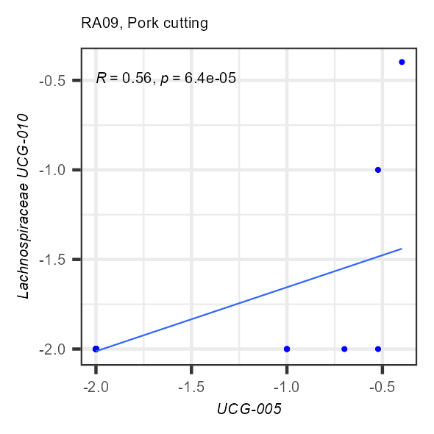


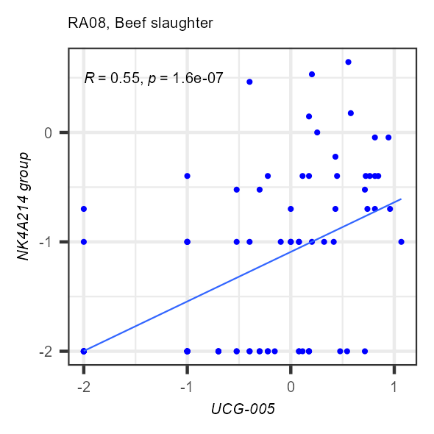

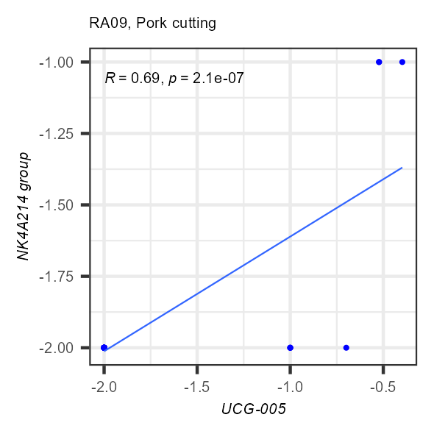


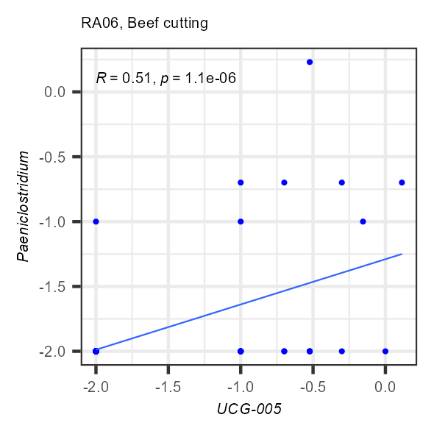

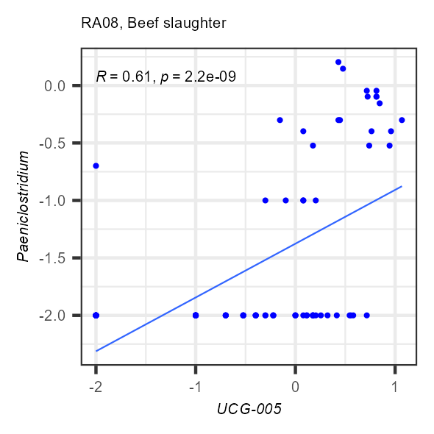

Supplement: Supplementary file 2 [file Data_Sheet_1.docx]
